# What is Learned from Longitudinal Studies of Advertising and Youth Drinking and Smoking? A Critical Assessment

**DOI:** 10.3390/ijerph7030870

**Published:** 2010-03-08

**Authors:** Jon P Nelson

**Affiliations:** Department of Economics, Pennsylvania State University, University Park, PA 16802, USA; E-Mail: jpn@psu.edu; Tel.: +1-814-237-0157; Fax: +1-814-863-4775

**Keywords:** youth, measurement of health, alcohol, tobacco, advertising, longitudinal models, econometrics

## Abstract

This paper assesses the methodology employed in longitudinal studies of advertising and youth drinking and smoking behaviors. These studies often are given a causal interpretation in the psychology and public health literatures. Four issues are examined from the perspective of econometrics. First, specification and validation of empirical models. Second, empirical issues associated with measures of advertising receptivity and exposure. Third, potential endogeneity of receptivity and exposure variables. Fourth, sample selection bias in baseline and follow-up surveys. Longitudinal studies reviewed include 20 studies of youth drinking and 26 studies of youth smoking. Substantial shortcomings are found in the studies, which preclude a causal interpretation.

## 1. Introduction

The health and welfare of adolescents is a major topic within health economics. Considerable attention has been paid in recent years to examination of risky behaviors by youth, such as smoking, drinking, drunk driving, drug use, unprotected sex, and crime [1–6]. These activities are often first undertaken prior to the age of nineteen, but can have important implications for longer-term health and welfare of adults. In this context, a possible definition of “risky behavior” is that the activity involves short-term benefits and potential longer-term costs. For example, the decision by a youth to engage in smoking or binge drinking has short-term payoffs that are both personal and social in nature. The longer-term costs can include adverse impacts on individual health, employability, longevity, marital stability, and other outcomes. Social external costs also can arise, either immediately or in the long run [7–9]. The ability of youth to weigh immediate benefits against potential adverse consequences is the subject of debate within economics and psychology. For economics, a starting point for modeling risky decisions is expected utility maximization whereby individuals weigh expected benefits against expected costs with exponential (time-consistent) preferences. In the “rational addiction” model due to Becker and Murphy [10–12], utility-maximizing individuals also incorporate interdependencies among past, current, and future consumption. Short-term benefits derived from current consumption are weighed against long-run costs, which include monetary and implicit costs associated with accumulated stocks of the addictive activity. A refutable hypothesis is that past, current, and *future* prices affect current consumption. Empirical tests of the rational addiction hypothesis have been carried out for several risky behaviors, including alcohol use, smoking, drug use, gambling, and obesity [13–17]. Several theoretical and econometric issues remain unresolved in the literature, especially when aggregate data are used [18–20].

Developmental psychology, as summarized by Fischoff [21], defines risk-taking as any action involving at least one uncertain outcome, where the outcome can be positive (winning a lottery) or negative (drug addiction). Hence, risk-taking is the deliberate choice of a risky behavior. The choice may be a single or infrequent behavior (drunk drinking) or a sequence of repeated behaviors (daily drinking). There should be voluntary choice at some point if an individual is to be described as taking risks, rather than just bearing them. According to Fischoff [21], the decision to undertake risky behavior is affected by an individual’s cognitive development (how people *think* about the world); affective development (how people *feel* about the world); and social development (roles that *others* play in people’s choices). For risky decisions, debate exists regarding the future orientation of youth compared to adults [18,22]. For example, some psychology studies report that consequences of risky activities involving social reactions are considered more heavily by teenagers than by adults, although the two groups are remarkably similar overall [23]. Peer pressure does not generally imply extreme forms of irrationality or purely emotional decision-making, but youth might react non-optimally to the intrinsic costs and benefits that they face. From an economic perspective, policies can be designed to manipulate incentives so as to better align perceived immediate benefits with longer-term social goals. Altering prices through the use of tax policy is one such example [24–30], but changing the regulatory or informational environment for risky decisions also can affect perceptions and incentives.

Now consider an environment in which the information used in decision-making is partly under the control of producers, such as the advertising messages and promotional campaigns used by producers of alcohol and tobacco. These messages might alter incentives to engage in underage drinking and smoking by glamorizing the activity, by altering perceptions of the risks involved with the activity or by altering perceptions of pervasiveness among peers and adults [31,32]. For example, the 1994 Surgeon General’s report [33] opines that “cigarette advertising appears to affect young people’s perceptions of the pervasiveness, image, and function of smoking” (p195). Psychologists use the term “false consensus effect” to describe individuals who perceive that their beliefs, choices or behaviors are relatively common [34]. The advertising messages also can be targeted to appeal to well-defined consumer groups or market segments according to dimensions such as age, gender, ethnicity, income, occupation, lifestyle, and past experience with the product. In the Bayesian learning model of addictive behavior due to Orphanides and Zervos [35], inexperienced individuals are initially uncertain of the potential harm associated with consumption of addictive products such as cigarettes. Each individual possesses a subjective belief or prior probability concerning his or her potential to become addicted, and this belief structure is updated with information gained from past consumption, peer behavior, schooling, advertising, and other marketing activities. In particular, advertising might alter the prior on the incidence of harmful addiction by changing youths’ smoker prototypes or their perceptions of the pervasiveness of smoking. Due to misperceptions, some experimenters with tobacco become addicted and may experience regret at a later point in life. Further, the misperception can be associated with social external costs. If accurate, the Bayesian model of addiction has implications for the content, placement, and amount of advertising and promotion that is permitted for risky products, especially those that involve youth or other inexperienced consumers.

On the other hand, advertising seeks to persuade and everyone knows it, even quite young children [36,37]. Consumers have reason to be skeptical of claims and images in advertisements because they recognize that ads represent producers’ self-interest, and sellers are aware that consumers are skeptical [38]. For this reason, consumers ignore or belittle many ads, but producers have an incentive to better match brands to consumer preferences and to compete with other sellers by informing consumers of this match. Consumers learn what brands to trust, so existing competitors and new sellers must work to overcome this trust, also known as brand loyalty. This essentially competitive process is most evident in the case of price advertising, where a series of economic studies have shown that bans of price advertising increase product prices [38,39]. More generally, by reducing information and restricting entry of new products and producers, advertising bans can entrench market shares and create market power for existing producers. Even in the case of persuasive advertising, such as that carried out for alcohol and tobacco products, there are self-correcting mechanisms in place. For example, advertisers sometimes engage in comparative (“less-bad”) brand advertising that also informs consumers of inherent risks associated with the product [38]. Advertising for well-known products with static markets (*i.e.*, mature products) rarely serves to increase industry sales, so a producer’s incentive often is to increase the number of its brands and direct its appeals for patronage to market segments. Some highly-advertised brands do better in the marketplace, but it is a fallacy to argue that successful advertising by one producer implies that all producers do better by advertising more [40,41].

However, advertising might be objectionable if it affects youthful consumers of restricted products, even if the products are legal for adults. Does mass-media advertising for alcohol and tobacco affect youth behaviors in a significant manner? How strong an influence is advertising and other marketing promotions? Most of the research directly bearing on these questions is found in survey studies carried out by psychologists and public health researchers. In particular, prospective cohort (longitudinal) studies are regarded as evidence of a causal relationship between marketing activities and drinking and smoking behaviors. Lovato *et al.* [42] argue that “longitudinal studies . . . capture what happens to individuals over time and can demonstrate whether individuals who differ in their exposure to advertising when they are not smoking, then differ in their future smoking behaviour” (p3). In a longitudinal study, a sample of youth is interviewed at least twice. During the initial or baseline survey, information is collected about each respondent’s use of alcohol or tobacco and his or her receptivity or exposure to mass media advertising and other marketing practices (branded merchandise, exposure in cinema and videos, *etc.*). All information on receptivity and exposure is self-reported, although various manipulations of these data are often performed such as combining responses to several survey questions to form an index. Data also are collected on possible covariates such as age, gender, race, ethnicity, family environment, parental education, school performance, and so forth. During one or more follow–up surveys, each individual’s drinking or smoking behavior is measured again. The follow-up survey usually is conducted one or two years later, but shorter and longer durations are possible. The behavior status at follow-up is typically analyzed using a logistic regression model, which computes the odds ratio of progression from, say, non-smoking to smoking status conditional on baseline receptivity and other covariates. It is argued that cross-sectional and time-series studies provide weaker “correlational” evidence because it is unclear if advertising exposure preceded the drinking or smoking outcomes [42,43]. Nevertheless, it is common practice for studies and reviews to cite these other methodologies if they provide supportive evidence [31,32].

Many longitudinal studies are based on Social Cognitive Theory, which incorporates aspects of social and cognitive development and learning [44,45]. Social cognitive theory suggests that a combination of environmental (social) and personal or cognitive factors influence behaviors (beliefs, attitudes, perceptions). Requirements for people to learn and model behavior are often divided into attention (attending to an advertisement); retention (remembering a brand); reproduction (initiating or intending to initiate product use); and motivation to adopt the behavior (net positive reinforcement). Advertising’s role is characterized as *symbolic modeling* [32], “in which the medium of observation is through mass media (such as television or movies) rather than face-to-face observation (such as a parent and child) . . . [however] audiences are conceived as complex and active agents in the person-media relationship” (p32). More specifically, expectancy beliefs are central to social cognitive theory, whereby individuals form *symbolic beliefs* or representations about the anticipated benefits and costs associated with a given behavior [46,47]. Using the technique of path analysis, elaborate models of expectancy theory have been constructed [40,47]. Compared to economic models, psychological models focus more on the process of decision making, which requires consideration of a wide variety of factors that might affect youthful decisions.

Several recent articles provide reviews of longitudinal studies of advertising and youth alcohol or tobacco behaviors [31,32,42,48–50]. These reviews conclude that advertising and marketing influence youth to use alcohol and tobacco, although the magnitude is sometimes labeled “modest”. However, the reviews provide simple narratives that focus on basic methodology and empirical results, especially results that conform to social learning theories. Assessments of the empirical model specification and statistical testing are frequently brief or absent. Assessments of the overall significance and magnitude are not reported in a summary fashion. Publication bias is ignored [51]. Despite the weaknesses in the studies and reviews, strong policy recommendations often are presented, such as calls for bans of all alcohol and tobacco advertising including passive advertising at sponsored sports events and similar venues. Given these past recommendations, the objective of this review is to provide a critical assessment of the modeling framework employed in longitudinal studies, the statistical procedures utilized, and empirical results achieved in such studies. In particular, I demonstrate that many longitudinal studies are seriously incomplete or ignore statistical problems and solutions that are well-known in econometrics, including issues of specification bias, measurement error, endogeneity, and sample selection bias. My conclusion is that the emphasis on advertising bans and similar regulations in the public health literature is misplaced. More effective policies need to be sought to deal with issues of youthful risk-taking associated with alcohol and tobacco.

It is of course possible for advertising to have a null or negative effect on the behaviors of adults and youth, and some longitudinal studies do report such results. Indeed, among economists, there is a long-standing belief that advertising does not have a large impact on aggregate product sales, either positive or negative [52]. There are three main reasons why advertising and other marketing activities may fail to influence behaviors. First, advertising can affect brand shares only with no effect whatsoever on initial purchase or eventual consumption, other than the choice of a particular brand. That is, the effect of advertising is purely redistributive even at the initial point in a consumer’s consumptive history. This is referred to as the “weak” or “predatory” theory of advertising [53]. Second, advertising can increase brand loyalty for some producers, which in turn increases the price that profit-maximizing producers find optimal. The increase in product price, which arises because the consumers’ demand function is rendered less elastic, reduces product demand. Additionally, due to advertising, consumer preferences or demand may be shifted or concentrated more in higher-quality (higher-priced) brands, so the indirect effect of higher prices can offset any direct effect of advertising. Third, advertising may raise costs and thereby increase product prices and reduce demand. In the context of an oligopoly, this is “a prisoners’ dilemma” equilibrium since all firms might be better off at a lower level of advertising. Although mixed results are reported, empirical research on the market for cigarettes demonstrate that restrictions on mass media advertising tend to lower consumption primarily by reducing price competition [54–56], so the direct effect of the restrictions is possibly benign. Overall, there are reasons to be skeptical that advertising plays a substantial role in youthful decisions to drink or smoke, over and above the choice of a particular brand.

The remainder of this review is organized as follows: Section 2 provides a brief summary of trends in adolescent drinking and smoking, using data for the US from the annual Monitoring the Future survey. Section 3 outlines some of the validation problems associated with empirical studies. Section 4 presents a summary of 20 longitudinal studies of youth drinking and 26 studies of youth smoking. The focus in this section is on model specification and testing, including critical analysis of measures used for receptivity and exposure to marketing and advertising. Tabular summaries are used to present the main features of the various studies and their findings. Section 5 analyzes longitudinal methodology with a focus on two advanced econometric issues, endogeneity and sample selection bias. Section 6 is a discussion of the findings, including a brief analysis of alternative methodologies presenting evidence on the effects of advertising bans for alcohol and tobacco. Section 7 contains the conclusions. Overall, the review finds that longitudinal studies of advertising and youth drinking-smoking behaviors contain significant econometric and statistical problems, which preclude a causal interpretation. Some suggestions are offered for improving the research in this area.

## 2. Trends in Adolescent Drinking and Smoking: Monitoring the Future

Systematic annual data on the prevalence of underage drinking and smoking in the US are collected and tracked by several organizations. This section relies on data from the Monitoring the Future (MTF) survey, which is an annual school-based survey conducted every spring since 1975. The most recent survey for 2009 includes information for about 45,000 secondary students in the 8th, 10th and 12th grades (ages 13–18). Selected information also is reported by MTF for full-time undergraduate college students (ages 19–22) and young adults ages 19–28 who are high school graduates. Alcohol data collected by MTF include any use in the past 30 days, daily use in the past 30 days, consumption of five or more drinks in the past 30 days, annual and lifetime use of alcohol. Various data also are collected on prevalence of drunkenness, type of beverage consumed, perceived risks and harms, and attitudes about disapproval and legality of underage drinking, heavy drinking, and drunkenness. Tobacco data collected by MTF include 30-day use, daily use, half-pack or more per day, annual and lifetime use of cigarettes. Various data also are collected on perceived risks of smoking, disapproval, use of smokeless tobacco, attitudes toward cigarette use, and ease of availability of tobacco products. For both products, subgroup data are reported by grade level, gender, race, *etc.* However, cautionary use of these data is necessary due to nonresponses (both individuals and schools) and inaccurate responses [57]. Other social processes, such as the stigmatization of tobacco use, also complicate interpretation of the data, leading to inferences that are at best tentative.

**Figure 1.**
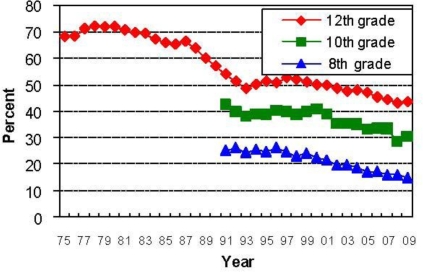
Trends in 30-Day Alcohol Use.

**Figure 2.**
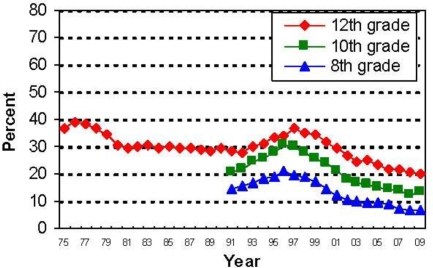
Trends in 30-Day Cigarette Use.

Table 1 and Figures 1 and 2 display the available information in the MTF surveys on 30-day prevalence for any use of alcohol and tobacco. Across grade levels, alcohol and tobacco use is high, but declining over time. Prevalence levels in the US also are below those in many other developed countries [58,59]. For alcohol, the trend for secondary students is toward lower levels of use, with an apparent slowing of the rate of decline in the mid-1990s followed by steady decline since the year 2000. One explanation for the decline is a higher minimum legal drinking age, which for the US has been 21 years in all states since the year 1989. For cigarettes, there is a sharp decline over time in smoking prevalence, although not always uniformly. The early 1980s was a steady period for prevalence as judged by 12th grade smoking, followed by a rise in the early 1990s. Since 1995, there has been a sharp decline in smoking at all age levels. Enactment of stricter regulations again played a role, but increased social stigma is probably important. However, smoking in the US is not federally illegal at ages younger than 18 as only the purchase of tobacco products is regulated. Some individual states have enacted laws making possession by a minor illegal or have increased the legal age for purchase. What else explains these trends? This turns out to be a surprisingly difficult question to answer, but higher real prices play an important role for tobacco products [60–62].

These trends also are of possible importance for longitudinal survey studies. First, the trend data show little change over short periods of one to three years, but there can be important changes over longer periods of time. Longitudinal studies that conduct a follow-up survey after more than three years may be capturing some of the trends revealed in the MTF data. Second, one possible way of explaining the trend in youth prevalence is to show that it is related to similar trends in adult behavior as shown by Cook and Moore [61], Gruber [62], and Nelson [63]. However, causality is unclear. The importance of adult behaviors may be that there is a direct effect if youth are influenced by adult prevalence. On the other hand, it can be that the adult and youth levels and trends reflect common underlying factors, such as a greater value placed on long-term health or similar responses to price changes. In either case, this reinforces the importance of accounting for a wide variety of factors in longitudinal studies, especially those covariates related to parental and peer behaviors.

## 3. Research Designs and Validity

It is useful to begin by thinking about an ideal research design, which is an experimental or randomized controlled trial. Suppose each youth in a random sample is to receive a specific treatment or “dose” of advertising, which is assigned by a random mechanism. Random selection and assignment of youth insures that the study groups are unbiased. Some groups may receive no treatment whatsoever, so there is a well-defined control group. The effect of the treatment could be measured on a before-after basis for each treated group, but the use of a control group can detect any “placebo” effects associated with the experiment. It is widely recognized that ethical considerations as well as practical problems restrict the use of experimental studies for advertising and youth drinking or smoking. In addition, no single experiment could capture the vast array of advertising and marketing methods used by producers. Any true experiment might underestimate the overall effects of advertising because only a limited number of key factors are studied at once [42]. Although some experimental studies of advertising have been conducted, they will not be reviewed here.

An alternative to a randomized trial is a “quasi-experimental” research design, where the treatment groups and comparison groups are not randomly assigned [64,65]. For example, econometric studies of advertising bans entail a statistical analysis of groups subject to complete or comprehensive bans, partial bans, and no bans of advertising. Both cross-section and time-series variation in the treatment may be present, so the data comprise a panel of observations. A “natural experiment” occurs if there is an exogenous source of variation across the groups, which might lead to changes in some important outcome. In his study of alcohol advertising bans, Nelson [66] argues that membership in the European Economic Union (EEU) constitutes a natural experiment for analysis of advertising bans and cross-country per capita alcohol consumption. EEU legal authority had the effect of changing advertising regulations independent of other country-specific influences on drinking. Other economic studies that might fall generally under the heading of quasi-experiments are reviewed in Section 6. An alternative quasi-experimental research design, widely used in psychology and public health, is a longitudinal survey study. Lovato *et al*. [42] argue that longitudinal studies can isolate the causal effect of advertising under the following conditions: (1) the advertising “treatment” must clearly precede the hypothesized effect; (2) behavior is measured at baseline and in one or more follow-ups; and (3) there are controls for possible confounding factors, such as age, gender, race, peer smoking, parental smoking, and so forth. Further, in most studies, a narrowly-defined cohort is selected based on age or school grade. This selection rules out some time-invariant confounders. Because the behavior measurements are essentially forward-looking, longitudinal studies reviewed here also are called “prospective cohort” studies.

Both longitudinal and econometric studies must address a number of general problems if empirical results are to be considered valid and possibly valuable for other purposes, such as policy design. Many problems arise due to the non-randomness of the treatment and comparison groups. If not addressed, these problems undermine any causal interpretation of the studies, so they are labeled “threats to validity” [65]. As defined by Campbell [67], *internal validity* refers to the confidence with which a causal relationship between two variables can be drawn. *External validity* refers to the confidence with which a presumed causal relationship can be generalized beyond the specific sample, setting, and time studied. Examination of internal validity is best carried out on a study-by-study basis, but the task of a review in part is to determine the external validity of a set of studies. The main threats to internal validity for longitudinal and econometric studies are as follows [65]:

1. Omitted variables: Personal characteristics of respondents and intervening events other than the “treatment” that provide alternative explanations for the outcomes. Omission of relevant explanatory variables results in specification bias, which is discussed further below.
2. Trends in outcomes: As explained above, there can be processes at work that are mainly a function of the passage of time per se, which may go undetected in the study.
3. Mismeasurement: A critical factor in longitudinal and econometric studies is the accurate measurement of advertising and marketing activities for alcohol and tobacco. This important threat is examined in detail below for longitudinal studies.
4. Misspecified variances: The significance of statistical tests is overstated if outcomes for some individuals are correlated or clustered, so the data have a group structure [64]. A number of treatments for clustered standard errors are now available.
5. Omitted interactions and paths: Omitted variables that capture differential effects by group, such as males and females, and omitted relationships that reflect more complex causal orders. As explained below, the terms for these influences in psychology are “moderated” and “mediated” effects.
6. Endogeneity: This term refers to the joint determination of outcomes. For example, many longitudinal studies determine youths’ baseline ownership of alcohol- or cigarette-branded merchandise and then measure the effect of baseline ownership on drinking or smoking outcomes at follow-up. After controlling for confounders, a significant positive relationship between ownership and outcomes is given a causal interpretation. However, in contrast to true experiments, ownership of the merchandise—or other exposure to advertising—is not randomly assigned, rather it is a choice on the part of the respondent. Hence, there is a strong possibility that ownership is endogenous, which requires a stochastic examination, and not predetermined or assigned in the experimental sense. As explained below, modeling of simultaneity is a common task in econometric studies, but this step is ignored in the longitudinal literature on youth drinking and smoking. As a result, empirical results in longitudinal studies are suspect due to simultaneity bias. Note that simultaneity is not an “economic” or “econometric” feature of the data; rather it arises due to use of a non-experimental research design. Its detection and measurement is critical to the internal validity of quasi-experimental research designs.
7. Selection bias: Selection can take many forms. For example, self-selection occurs if respondents can opt out of the survey and their participation decision is based on characteristics that also are relevant to drinking or smoking outcomes, but are unobserved. As shown by Heckman [68], self selection creates specification bias for the empirical relationship. The crucial detail is that the sample is no longer random and there are omitted variables associated with the participation decision.
8. Sample attrition: The differential loss of participants from different groups, such as the failure of minority students to participate in the follow-up survey at a rate comparable to non-minority students. Both selection and attrition threats are discussed in detail below.

With the exception of endogeneity, all of the threats to internal validity are well-known statistical problems in the psychology and public health literatures, although individual studies may fail to fully recognize or deal with some threats. It is a special feature of many econometric studies that they grapple with endogeneity issues, where several statistical techniques are available. In the remainder of this review, the threats are taken up in conventional order, but readers with a strong background in econometrics might desire to read Section 5 prior to the rest of the review. Many details are examined for model specification and measurement, but it cannot be emphasized too strongly that the single-most important contribution of health econometrics is its focus on endogeneity issues.

## 4. Specification and Estimation of Longitudinal Models: Alcohol and Tobacco

### 4.1. Model Specification: Specification Bias and Measurement Errors

This section reviews the model specification used in 20 longitudinal studies of youth drinking and 26 studies of youth smoking. Overall, there are a number of common features of the studies, which allow cross-study comparisons and generalizations. However, the two groups of studies employ slightly different models, especially for advertising receptivity and exposure. I first examine the model specifications used in studies of youth alcohol behavior and summarize the difficulties associated with the measures of advertising in these studies, especially the consistency of empirical results across studies. Second, I examine the model specifications used in studies of youth smoking behavior, and offer critical assessments of these studies. In both cases, there are numerous empirical estimates that are null or statistically insignificant that tend to be ignored in other reviews and policy discussions.

Specification errors arise when an empirical model omits a relevant covariate (omitted variable bias) or when an empirical model includes an irrelevant variable [69]. In the first instance of under-specification, the least-squares estimator of the remaining variable(s) is biased, with the direction of bias depending on the correlation between the omitted variable and the included variable and the correct sign of the omitted variable on the outcome in question. Suppose “risk-preferences” of survey respondents are positively correlated with receptivity to marketing, and risk-preference has a positive effect on drinking and smoking behaviors. If risk-preference is omitted as a covariate or unobserved, a positive correlation and a positive (but omitted) sign impart a positive bias to the receptivity variable [70]. The measured effect of marketing is overstated. The size of the bias is determined by the effect size of the omitted variable (risk) and the partial effect of risk on receptivity, *i.e.*, the risk-adjusted effect of receptivity. Hence, it is crucial in an empirical study that all “important” variables are included, especially those that are more highly correlated with the explanatory variable that is the focus of the investigation. Potentially, this list of variables is quite long. In a multivariate context, the bias can be transferred to other covariates depending on the pattern of correlations among the included variables. Only in the unlikely case where all covariates are uncorrelated (orthogonal regressors) is the bias avoided. The variance-covariance matrix for the included variables is unbiased, but larger in magnitude (less efficient). Further, including an irrelevant variable does not result in bias for the other variables, although the least-squares estimator is not as efficient. Because there is always uncertainty regarding the “correct” model specification, a number of statistical tests and diagnostics have been developed, including tests for omitted variables, functional form, structural change, and data outliers [69,70]. More generally, it is common practice in econometrics to present results for several different specifications or estimation methods, which tests less formally the robustness of the results for omitted variables [69]. This is referred to as a robustness check or “sensitivity” analysis. As discussed below, both formal and informal specification tests are rarely conducted in longitudinal studies.

The second problem discussed in this section is measurement error associated with variables for advertising receptivity and exposure (errors-in-variable problem). Ideally, an advertising or marketing measure should satisfy three criteria. First, it should represent accurately the forces that influence a decision by a youth to consume alcohol or tobacco, insofar as these decisions are affected by advertising and marketing. Second, all relevant advertising and marketing variables are included in the final regression (otherwise there is specification bias). Third, the advertising variables in the model should be related in some manner to actual or anticipated public policies, such as broadcast advertising bans or restrictions on the contents of advertisements and promotions. In the first instance, mismeasuring the advertising variable renders this explanatory variable stochastic and hence correlated with the error term in the regression. The least-squares estimator of the advertising coefficient is biased and inconsistent, *i.e.*, the problem is not solved by increasing the sample size [69]. In general, the bias is toward zero, with the magnitude of the bias depending on the how much variation there is in the true variable measured without bias and the variation in the measurement error [70]. In the multivariate case, estimators of all included variables can be biased and inconsistent. However, the direction of the bias can go either way and is generally difficult to determine. Econometric procedures for dealing with errors-in-variable bias include use of instrumental variables, but traditional methods applied to survey data present special problems [71]. Latent variable models have been widely used in health economics [72], with several indicator variables that are related linearly to unobserved true values of the mismeasured variable. However, instrumental variable estimation is a special case, which avoids strong assumptions regarding measurement error variances [69].

In addition to measurement and specification errors, many of the receptivity-exposure measures present special problems for assessment of public policies toward alcohol and tobacco advertising. Most measures in question are not demonstrated to be related to or even correlated with actual advertising exposure or with any actual public policy [73]. This is in sharp contrast to econometric studies that attempt in various ways to evaluate the effects of advertising expenditures, broadcast advertising bans, and other regulatory policies (e.g., price advertising bans, billboard bans, warning labels). Because the receptivity measures in longitudinal studies tend to be broad and amorphous, it is difficult to say (or even guess) how receptivity might change in light of a particular public policy. Hence, as a crude policy application, longitudinal studies usually are led to recommend complete bans of all forms of alcohol and tobacco advertising, but this is not a statistical application or even logical extension of the models. It is not possible to simulate the consequences of the proposal to see if the estimated results are reasonable. As a result of these problems, most longitudinal studies are devoid of policy implications as that term is commonly understood by economists.

A final issue here concerns the nomenclature applied to the right-hand side (RHS) of regression equations. In econometrics, RHS variables (the “X” variables) are usually referred to as regressors, covariates, independent variables, or explanatory variables. The outcome or response is the regressand or dependent variable (the LHS “y” variable). Other disciplines frequently employ the term “confounders” or “extraneous” variables to refer to RHS variables other than the variable(s) that are the focus of the study. However, in psychology, an important distinction also is drawn between RHS variables that are moderators and mediators of a focus variable [74]. Briefly, a “moderator” variable is third variable (z) that interacts with a variable x so as to affect the relationship of x and y. This moderating effect can be accounted for by including z in the regression and an interaction term between x and z, given by the variable xz. The relationship between x and y now depends on the level of z. Hence, excluding the interaction variable from the regression is a specification error and could result in specification bias of some magnitude. Since multicollinearity also is an issue here, tests for statistical significance generally require examining the joint significance of the coefficients for x, z, and xz. In econometrics, moderating effects are accounted for in the same fashion or by choice of functional form for the regression (double-log, semi-log, translog). A “mediator” variable is a variable that both causes y and is caused by x, so in a path analysis diagram there is direct path from x to y and an indirect or intervening path through which x causes z and z causes y. Note that the mediated relationship is theoretical as opposed to a moderating relationship, which can be treated as a purely statistical problem. Mediating variables are not usually considered as endogenous variables in the psychology literature. As long as the indirect relationship or amount of mediation between x and z is not of major importance, then estimation by multivariate least-squares is straightforward. However, this precludes a complete causal or structural interpretation, which requires estimation by path analysis or similar models. Several of the studies reviewed below report results for interaction variables or use multilevel hierarchical or path analysis models for mediation effects. Complete examination of these models is beyond the scope of this review, although some critical comments have been offered elsewhere [40,51].

### 4.2. Alcohol Advertising: Model Specification in Twenty Studies

Twenty studies were determined to meet the following criteria: (1) a longitudinal study with baseline and follow-up sample(s) of youth or young adults; (2) one or more drinking behaviors as outcomes (drinking onset, frequency, binge drinking, *etc.*); (3) one or more advertising, marketing, promotional, brand recognition or receptivity measures as covariates, determined at baseline (one exception); and (4) the study uses a multivariate statistical procedure such as logistic regressions. Some studies noted below use the same sample of respondents, but analyze the data in different ways. For the most part, the studies were identified by using previous surveys and searches on MEDLINE, PsycINFO or Google, and reference lists in more recent publications. Experimental, cross-sectional, and time-series or econometric studies are excluded from this review. Also excluded are studies of drinking intentions. Three previous reviews covered 16, 9 and 10 studies, respectively [48–50].

The 20 studies are summarized in the Appendix [75–94]. Fourteen of the studies are for the US, including four nationwide studies. The other countries studied are Belgium (1 study), Germany (2), and New Zealand (3). Some studies use several waves of a continuing survey, such as the New Zealand studies. Some studies use identical or very similar samples: there are two identical nationwide US studies [85,92]; two studies of South Dakota middle school students [77,79]; two studies of middle school students in New Hampshire and Vermont [84,88]; and two studies of German youth [81,82]. A common theme in these overlapping studies is emphasis on different methods of alcohol advertising and marketing. For example, McClure *et al.* [84] use the New Hampshire-Vermont sample to study the effect of alcohol-branded merchandise on drinking onset of youth who were baseline nondrinkers. The same sample and drinking outcome is used by Sargent *et al.* [88] to examine the effect of exposure to alcohol portrayals in movies. Neither study mentions or includes the data on the other promotional method. Both models are therefore misspecified and potentially biased. This statement also applies to other overlapping studies. The age range for respondents in the studies includes youth who generally are 16 years or younger, but several studies also include young adults [75,76,89]. In some cases, the empirical analysis is conducted using subsamples by age or gender [75,78,80,89]. Although attrition and completion rates in the surveys are difficult to determine exactly, most of the surveys indicate a completion rate from baseline to follow-up of about 70% or better. The exception is the survey used by Snyder *et al.* [89], which had sample sizes in four waves of 1,872, 1,173, 787, and 588 respondents.

Multiple outcome measures are reported including drinking onset by baseline nondrinkers, any alcohol use, average amount per occasion, frequency of consumption, binge drinking, and other measures such as maximum amount. A difficulty here is that drinking onset tends to cover any use, which does not necessarily imply continued or frequent use of alcohol. The same problem applies to current or “regular” drinking as an outcome, usually defined as any alcohol use in the 30 days prior to the survey. All outcome measures rely on self-reports by the respondents, but it is difficult to determine if any cross-checks are included in the survey questions. The studies cover a range of outcomes and use different statistical models, which makes quantitative summaries more difficult to achieve. The exceptions are those studies that examine drinking onset and behaviors by baseline nondrinkers and employ logistic (or log-link) regression models.

In order to isolate the effects of alcohol advertising and promotion on youth drinking behaviors, it is necessary to control for important covariates or confounders. Lists of included covariates are reported in the last column of the Appendix table. Substantial diversity is revealed in the lists. In many cases, social learning theory is cited as a basis for the model specification for advertising, but there is little discussion of the important covariates that permit a valid test of the effect of advertising in this theory. Most model specifications are rather ad hoc, and are not guided by a well-defined theoretical model. A few studies are unclear as to the final list of covariates [90,93]. Some studies include only a few basic demographics [76,77,87,91] or exclude important variables such as parental or peer drinking [75,76,78,86–89,91,93]. Measures of risk-taking or impulsiveness are included in some studies, but this is far from universal [77,79,81,83–86,88,92]. Smoking status is a covariate in five studies [80,81,84,88,91]. Interaction variables for moderating effects are employed in only three studies [80,86,90], while four studies estimate structural models [76,81,92,94].

What is required in order for the studies to pass an internal validity test? First, the studies need a better rationale for the model specification. A starting point is provided by Ellickson *et al.* [79], where 15 covariates are divided into several broad categories, including social influences (e.g., peer drinking), social bonds (religiosity, parental monitoring), attitudes and behavior (impulsivity), and demographics (gender, *etc.*). In Henriksen *et al.* [83], the categories are social influences (peer drinking), psychosocial risk factors (school performance), and demographics. Classifications such as these serve to indicate the presence or absence of important explanatory variables and facilitate comparisons across studies. Consulting other surveys in this area would be helpful, such as the review of familial influences by Avenevoli and Merikangas [95]. Second, more extensive testing of model specifications should be carried out in the form of a robustness check or sensitivity analysis. This is a standard practice in econometrics, but almost never included (or reported) in longitudinal studies. Formal methods of model specification such as Hendry’s general-to-specific procedures should be considered [96]. Correlations or variance inflation factors among the covariates are seldom reported to see if there is extensive duplication of information due to multicollinearity. Full reporting of empirical results also is encouraged as some studies lack p-values (or standard errors) or fail to report coefficient estimates for all covariates [78,81,83,85–87,90,93].

Third, some studies use more than one market area and need to consider the inclusion of market-area variables, such as beverage prices, regulations, average income, and other aggregate variables. This is standard practice in econometric studies, including those based on survey data such as Markowitz and Grossman [97] for alcohol regulation and Czart *et al.* [98] for smoking regulation. Two nationwide longitudinal alcohol studies for the US use samples of over 6500 youth [85,92], but neither includes location-specific variables that might be important for youthful decisions. Other studies use multiple market areas [89] or cover broader geographic regions [77,79,81,82,84,88,91], which also might require additional data. Aggregate variables also may be required for studies in which the follow-up survey is more than three years after the baseline survey, such as Casswell *et al.* [75]. The necessity of controlling for the market environment does not seem to have been considered by survey researchers. The importance of prices for youthful drinking and smoking speaks against this omission in survey studies. This issue is discussed further in Section 6.

### 4.3. Alcohol Advertising Studies: Measures of Advertising and Promotion

The information in the Appendix table reveals a wide variety of measures of advertising and other forms of promotion for alcohol. A diverse set of empirical results also is revealed. Upon close examination, there are studies with negative, null, and positive results for advertisements, sometimes in the same study [75,79,87,90]. Results for some variables, such as TV exposure and other mass media, are especially fragile. In Table 2, I first provide a narrative summary of the advertising measures, making note of the range of measures, studies that restrict the variables to one or two covariates, and other specification and measurement errors. Second, I provide a quantitative summary of the results for a selected group of 12 studies that estimate a logistic (or log-link) model.

There are several critical points to make about this information. First, there is little replication of measures across studies, making assessment more difficult. For example, six studies use general TV viewing habits as a covariate, but this is unrelated to exposure to alcohol ads and might be a surrogate for personality traits. Four studies use ownership of an alcohol-branded item (ABI) as a covariate and five studies use movie portrayals of alcohol. However, there is overlapping information in the sets of studies. Two German studies [81,82] use the same sample to examine two different measures of movie exposure. McClure *et al.* [84] and Sargent *et al.* [88] use the same New England sample to study the effects of ABIs and movies, respectively. McClure *et al.* [85] and Wills *et al.* [92] use the same nationwide US sample to study ABIs and movies, respectively. Hence there is less independent information than might be apparent from Table 2. Further, these studies are misspecified due to the omission of the other promotion variables; that is, there is no statistical rationale for regressing alcohol outcomes on ABIs in one study and omitting it as a covariate in a related study of movie exposure. In general, this will bias the advertising coefficient in each study toward a larger positive value. The same critical comments apply to other overlapping studies, including the studies by Collins *et al.* [77] and Ellickson *et al.* [79] for South Dakota students.

Second, given the variety of advertising measures in Table 2, it is difficult to understand why so many studies severely restrict the number of measures or fail to validate the measures with more complete models. A few studies experiment with general indexes such as advertising receptivity and liking/awareness of ads and brands. For example, in Henriksen *et al.* [83], a composite index of receptivity to alcohol marketing is based on survey responses for: (1) “having a favorite alcohol advertisement”; and (2) “owning or wanting to own alcohol promotion items”. Based on responses, individuals are divided into minimal, moderate, and high receptivity groups. Henriksen *et al.* [83] find statistical associations between high receptivity and drinking onset and current drinking, but the model is poorly specified and receptivity is not a robust variable. As pointed out by Heckman [73], it is important to control for all other plausible factors in order to establish a causal relationship, but Henriksen *et al.* [83] exclude all familial variables. Further, it is possible that marketing receptivity is capturing unobserved attitudes and preferences toward drinking. That is, survey respondents who are more likely to drink would be more likely to be classified as high-receptivity individuals, all other things held equal. Henriksen *et al.* [83] provide evidence that alcohol-marketing receptivity is positively associated with variables for individual risk-taking, lower grades, perceived prevalence, and perceived approval. Hence, high receptivity may simply be capturing these and other underlying attitudes and preferences. A plausible conclusion in this and other longitudinal studies is that adolescents with a greater preference for risk or higher perceptions of drinking prevalence/approval are more likely to drink. In McClure *et al.* [85], ownership of an ABI is positively associated with variables for sensation-seeking, rebelliousness, and peer drinking. Including an interaction variable between these preference variables and ABI ownership would be useful to determine if the effect of ABI-ownership is greater for certain groups of youth. Only a few of the 20 studies have experimented with interaction effects [80,86,90]. Similar comments apply to studies that measure liking of ads.

Third, evidence on brand recognition or brand approval is not clear evidence regarding the general effects of advertising-marketing on youth drinking behaviors or evidence of a causal effect of advertising on youthful drinking. It is not surprising that youth (as well as adults) have a favorite brand or are able to recognize brands in favorite advertisements. There are informational advantages to new consumers to associate their consumption with a well-known brand, so economic incentives work in favor of choosing a brand, especially a well-known brand. Well-known brands also tend to be highly advertised. However, brand-related variables do not provide convincing evidence of a causal link with youthful choices. It would not be surprising that individuals who watch lots of broadcasts of professional football or auto racing also own more branded merchandise related to those sports. It is not logical to argue that ownership of the merchandise *caused* them to watch the broadcasts or express loyalty for a particular team. Brand loyalty is different than a causal effect of advertising.

Given these shortcomings, a final issue is to examine the studies for consistency of empirical results, which is a simple test of *external validity*. Table 3 presents a summary of the results for 12 studies that use a logistic or log-link model, which draws on my comprehensive meta-analysis of longitudinal studies [51]. Results are summarized for the odds ratio for advertising-marketing variables for drinking onset and other drinking behaviors (frequency, amount, binge drinking, *etc.*). Advertising and marketing variables can be divided into four categories: (1) mass media (TV, magazines, billboards, *etc.*); (2) promotion portrayals (ABIs, movies, videos); (3) other media exposures (in-store displays, concessions); and (4) attitudinal or subjective measures (liking of ads, brand recall, *etc.*). In Nelson [51], I demonstrate that publication bias is a substantial problem in longitudinal studies, which is ignored by prior systematic surveys [48–50]. Publication bias occurs when the publication of empirical results depend on the direction, significance, and magnitude of the results [99,100]. Due to emphasis on statistical significance, published studies are likely to be skewed toward larger effects. As a consequence, the published studies comprise a biased sample, so the results of a literature review or meta-analysis can be misleading. Hence, it is important to take note of insignificant results in longitudinal studies of advertising and youth alcohol behaviors.

In Table 3, there are 63 estimates of the effects of advertising and promotion on adolescent drinking. A variety of drinking behaviors are examined, including onset of drinking, maintenance by baseline drinkers, drinking amounts by beverage, frequency, and binge drinking. Only 21 of 63 estimates (33%) are statistically significant at the standard 95% confidence level. This does not support recommendations for bans of advertising. For drinking onset, only one estimate for mass media is statistically significant. For other drinking behaviors, only 5 of 14 estimates for mass media are statistically significant, but 4 of these estimates are from the same study by Stacy *et al.* for Los Angeles youth [90]. Ten of 15 estimates for promotional portrayals, including ABIs and movies, are significant, but several z-statistics are close to the lower limit of 2.0. The distribution is right-skewed and several studies produce point estimates that are outliers (more than two standard deviations above the mean), especially the estimates for movie displays in [81] and [82]. Several estimates for ABIs in [77,80,85] are close to this cutoff.

Finally, in Table 3, there are 18 estimates of the effect of TV viewing on drinking onset and drinking behaviors, which are significant in 7 cases and insignificant in 11 cases. Both estimates for magazines are insignificant. There are 10 estimates for ABIs, 6 of which are significant and 4 are insignificant. A similar problem exists for studies of movie displays. Except for one estimate for in-store displays, none of the 10 estimates for other promotions are significant. There are 8 estimates for subjective “liking of ads”, “awareness of ads” and “self-reported ad exposure”. Only one estimate is statistically significant. None of the 7 estimates for brand awareness are statistically significant. These results raise questions of what exactly is being captured by more objective measures of marketing exposure. One possibility is that youth who are predisposed to drink for unobserved reasons also are attracted to advertising and promotion of alcohol. This means generally that the models used in longitudinal studies should treat measures of advertising and marketing as endogenous variables, and not predetermined or exogenous variables. This issue is addressed below.

### 4.4. Tobacco Advertising: Model Specification in Twenty-Six Studies

Twenty-six tobacco studies were determined to meet the following criteria: (1) a longitudinal study with baseline and follow-up sample(s) of youth; (2) one or more smoking behaviors as outcomes (smoking susceptibility, onset, regular smoker, *etc.*); (3) one or more advertising, marketing, promotional, brand recognition or receptivity measures as covariates, determined at baseline (one exception); and (4) the study uses a multivariate statistical procedure such as logistic regressions (one exception). Excluded are longitudinal studies that use descriptive methods for analysis or which examine smoking intentions, anti-smoking media campaigns, young adults exclusively, or smoking regulations. Some studies noted below use the same sample of respondents, but analyze the data in different ways. For the most part, the studies were identified by using previous surveys and searches on MEDLINE, PsycINFO or Google, and reference lists in more recent publications. Experimental, cross-sectional, and time-series or econometric studies are excluded from this review. Three previous systematic reviews of longitudinal studies covered 16, 9, and 13 studies, respectively [32,42,101].

The 26 studies are summarized in the Appendix [81,102–126]. Nineteen studies are for the US, including two nationwide studies. The other countries studied are Australia (2 studies), Germany (2), Mexico (1), Spain (1), and the United Kingdom (1). Some studies use several waves of a continuing survey, such as the studies using the California Tobacco Survey (CTS). Some studies use identical or very similar samples: there are three studies for Massachusetts [104,105,117]; two studies for California for 1993–1996 [107,114]; three studies for California for 1996−1999 [109,115,116]; two studies for New Hampshire and Vermont [108,120]; and two studies for Germany [81,122]. Common themes in these overlapping studies are emphasis on different tobacco advertising methods or different smoking outcomes. For example, Pierce *et al.* [116] use the 1996−1999 CTS to examine the effect of advertising receptivity on smoking susceptibility, controlling for the covariate “curiosity about smoking”. Virtually the same sample is used by Distefan *et al.* [109] to examine the effects of smoking by a favorite movie star on smoking onset, but curiosity is omitted as a covariate. The use of overlapping samples means the amount of independent information in the table is less than what is apparent. In some cases, the empirical analysis is conducted using subsamples by age, gender, race, country of birth, and parenting style [106,111,112,115,125]. These studies suggest that heterogeneity of respondents is very important for measuring the influence of advertising on smoking behaviors. Across the 26 studies, the range of ages for respondents at baseline is about 10–15 years (grades 6–9), but one study [111] includes older respondents and one [123] uses younger respondents. Attrition and completion rates in the surveys are difficult to determine exactly, but most of the surveys indicate a completion rate from baseline to follow-up of about 65%. In several of the CTS studies, the rate is below 50% [107,111]. One study [106] had a completion rate of 100% but the surveys are separated by only four months, suggesting this study might be better treated as a cross-sectional study. Several studies use surveys separated by 4 to 6 years, which raises issues of confounding due to trend effects.

The outcome measures in the studies include regular smoking, onset of smoking, experimenting with smoking, susceptibility to smoking, and a smoking index. A few studies use two or more outcomes [117,123,126]. Except for regular smoking and experimentation, these measures present interpretation problems. Onset measures combine experimenters, occasional smokers, and regular smokers, while susceptibility measures combine susceptible nonsmokers with experimenters and (possibly) regular smokers. The ordinal smoking indexes combine all individuals from non-susceptibiles to regular smokers, but it is unclear if a linear scale is appropriate for this task. It is sometimes argued that using smoking susceptibility as an outcome is a “more sensitive” measure than actual smoking [119], but this ignores the possibility that the two groups (susceptibiles and smokers) might have fundamentally different responses to advertising stimuli. Unfortunately, this issue has not been addressed within the confines of a single sample of respondents.

In order to accurately isolate the effects of tobacco advertising and promotion on youth smoking behaviors, it is necessary to control for important covariates or confounders. Lists of included covariates are reported in the last column of the Appendix table. Substantial diversity is revealed in the lists. Most studies include basic demographic information (age, gender, race/ethnicity) and most studies include variables for parental and peer smoking, but there are exceptions [111,117,119]. Measures of risk-taking or impulsiveness are included in some, but not all, studies [81,104,108,112,120,121]. A number of studies include interaction variables that test for moderator effects, with mixed results [102,107–109,111,113–116,119,122,125]. Some studies, such as Biener and Siegel [104], use simple bivariate regressions to select the variables in their final model, but fail to consider interactions between, say, receptivity and parental smoking and fail to examine multicollinearity issues. Several studies test for mediation effects by using multilevel, path analysis or other structural equation models [81,105,119–121], but these studies treat advertising as an exogenous variable and do not test for possible endogeneity (see below). In a number of cases, full results for covariates are not reported [81,102–104,108,110,113,115,117,118,126]. In Pierce *et al.* [116], the variable for “curiosity about smoking” is included as a possible covariate for experimentation and susceptibility to smoking. Past curiosity about smoking at baseline is a strong predictor of experimentation and susceptibility at follow-up. However, including curiosity as a regressor leads to *insignificant* results for receptivity to tobacco advertising in both regressions. This raises an important issue of omitted variable bias in other studies, since this is the only study that considers curiosity as an intermediate goal of advertising. Pierce *et al.* [116] argue that curiosity appears to be an antecedent to susceptibility to smoking, but it is unclear why only higher levels of receptivity are associated with curiosity in a cross-sectional regression. As discussed below, “curiosity” as a personality trait could be used to construct an instrumental variable estimate of the advertising-marketing covariate [64,127].

What is required in order for the studies to pass an internal validity test? In many cases, social learning theory is cited as a basis for the model specification for advertising, but there is little discussion of the important covariates that permit a valid test of the effects of advertising in this theory. Most model specifications are rather ad hoc, and are not guided by a well-defined theoretical model. First, the studies need a better rationale for model specification. Several studies provide categorical summaries of the covariates, such as that in Alexander *et al.* [102] where variables are classified according to “attitudes”, “knowledge”, “social and personal factors” and “usage of other drugs”. For most part, longitudinal studies simply present a list of covariates with little justification, although sometimes there is a supporting cross-sectional study that delves into the specifics of the survey instrument and sampling procedures [108,128]. Second, more extensive testing of model specification should be carried out in the form of a robustness check or sensitivity analysis (see above on alcohol studies). Third, some studies use multiarea samples, such as two nationwide surveys for the US [110,121]. More generally, all of the tobacco studies employ broad geographic areas, where prices, economic conditions, smoking regulations, health information and costs, and other variables can differ across jurisdictions. Studies for states as large as California, Massachusetts, and North Carolina need to consider including variables that control for the market environment as well as the social environment. This omission is a common theme for all alcohol and tobacco studies. An alternative approach, widely used in econometrics, is a fixed-effects panel model, with binary indicator variables for schools, jurisdictions, states, *etc.* [64,69,70]. Unobserved heterogeneity is captured by the fixed-effect intercept for each group in the panel. No longitudinal study has incorporated this methodology.

### 4.5. Tobacco Advertising Studies: Measures of Advertising and Promotion

Reflecting restrictions on tobacco advertising, longitudinal studies have relied on fewer measures of advertising and promotion compared to similar studies for alcohol. Allowing for multiple measures in some studies, the Appendix table indicates the following measures have been used: receptivity index, movie-smoking exposure, general TV viewing, knowledge of brands, approval/favorite cigarette ads, ownership of a cigarette-branded item, and magazine exposure. None of these measures is validated in the sense that it is shown to be related to actual advertising campaigns or public regulations that have affected tobacco advertising. General measures, such as TV viewing, are especially suspect. Receptivity to tobacco marketing has been used often as an advertising measure, but it is a rather broad and amorphous index. As originally proposed by Pierce *et al.* [114], receptivity captures exposure to a communication and a cognitive response that indicates an understanding of the communication. Survey respondents are asked three questions in order to determine their position on a receptivity scale, such as: (1) “Do you own or would you be willing to use a cigarette-branded item (CBI), such as a t-shirt?”; (2) “What brand of cigarette was most advertised in ads you have recently seen on billboards or in magazines?”; and (3) What is the name of the cigarette brand of your favorite advertisement?” Depending on the study, respondents are considered to be “highly” receptive if the first question is answered affirmatively; “moderately” receptive if the third question is answered with a specific brand, but they answer the first question negatively; and “low” receptivity if the second question is answered, but the respondent does not name a favorite ad or own a CBI. Hence, the receptivity index is: minimal (no brand, no favorite ad, no CBI); low (brand, no favorite ad, no CBI); moderate (brand, favorite ad, no CBI); and high receptivity (willing/has CBI).

There are several things to note critically about receptivity indexes. First, receptivity is largely about brand recognition and not about exposure to different levels of advertising. Hence, it is possible that receptivity merely tests the “weak” theory of advertising or demonstrates that advertised brands are recognized by smokers. No causal interpretation should be assigned to this relationship. Second, receptivity measures have not been validated by showing they measure exposure to actual cigarette advertising campaigns. The terminology used in longitudinal studies confuses personal preferences with advertising exposure [73]. Third, respondents with high receptivity may differ in important but unobservable ways from those with lower levels of receptivity. It should not be assumed that respondents who wear a branded t-shirt (or are willing to wear one) are in all ways identical to those who do not, except for the observable covariates in the regression equation. Fourth, as discussed below, receptivity and indeed all measures of advertising and marketing involve choices on the part of the respondent. Each respondent chooses to own or not own a CBI. Each respondent chooses to view more or fewer R-rated movies. The choice process is fundamental to testing of theoretical models of the effects of advertising and marketing on youthful behaviors regarding alcohol and tobacco. Longitudinal studies in psychology and public health ignore this part of the problem and treat receptivity as a purely exogenous “treatment”, and thereby seek to estimate a “dose-response” relationship. The level of dosage is not randomly assigned to each respondent, but is rather chosen by the respondent. Hence, in order to demonstrate causality, a detailed model of the choice process and the accompanying incentive structure must be sought [73].

Given these shortcomings, the final issue is to examine the studies for consistency of empirical results. Table 4 presents a summary for 18 studies that use a logistic or log-link model. Results are summarized for the odds ratio and 95% confidence interval for groups of studies by outcome: regular smoker, onset of smoking, experimentation, susceptibility, and smoking indexes. Overall, there are 63 empirical results reported in Table 4, which are insignificant in 32 cases (51%). Low receptivity is insignificant in 6 of 6 cases and moderate receptivity is insignificant in 7 of 10 cases. High receptivity is insignificant in 3 of 10 cases. Overall, receptivity is insignificant in 16 of 26 cases (62%). The lack of robust results is an indication of theoretical weaknesses or evidence of measurement errors. The results for high receptivity may reveal only an association between brand loyalty and smoking behaviors. The results are even poorer for general TV exposure. There are 8 results for TV-viewing, which are significant in 2 cases and insignificant in 6 others. For movie exposure, the results are insignificant in 8 of 22 cases. The distribution is right-skewed and some coefficient estimates are outliers (*i.e.*, more than two standard deviations above the mean), especially values for TV viewing in [110]. It might be argued that the evidence is stronger for high levels of receptivity or exposure, but it also might be the case that youth who wear cigarette-branded t-shirts, watch lots of TV and videos, and go to lots of R-rated movies are different in fundamental ways from youth who moderate these behaviors. Finally, no study includes more than two measures of advertising and marketing, so there is omitted variable bias in all studies. One study [112] merely reports separate regressions for movies and TV. Overall, this is a generally weak set of results that fail to support either a causal relationship between advertising and youth smoking or the implied public policy of advertising bans.

As shown in the Appendix, there are ten longitudinal studies of movie-smoking exposure and youth smoking behaviors for the United States, Germany, and Mexico [81,108,109,112,120–125]. A review by Charlesworth and Glantz [129] of the movie-exposure literature argues that there is “strong empirical evidence that smoking in movies increases adolescent smoking initiation” (p1516). However, Omidvari *et al.* [130] point out that smoking prevalence in US movies is no more common than in real life (23.2% *vs.* 24.8%, respectively). They reviewed 447 movies that depicted contemporary American life in the 1990s (R-rated, 193; PG13-rated, 131; and PG-rated, 123). The movies are chosen from the top 10 movies on the weekly box office charts, so they are in general popular films that are seen by many viewers. Overall, the depiction of smoking in movies is more prevalent for men, antagonistic characters (“bad guys” and “villains”), lower socioeconomic class (SEC) characters, whites compared to non-whites, independent movies rather than studio films, and R-rated movies. In particular, smoking prevalence in R-rated movies is higher than the general US population for both studio films (30.5%) and independent productions (50.6%). The majority of R-rated films portray smokers as white males, lower-middle SEC, and antagonists. Much has been written negatively about product placement of cigarettes in cinema. Omidvari *et al.* [130] point out that, contrary to previous reports, “if there is a conscious attempt to influence public smoking habits, it is being orchestrated by independent movies, and not by Hollywood” (p751). These conclusions are not unexpected. Independent producers (“entrants”) must differentiate themselves from established producers (“incumbents”) by making more anti-establishment films that appeal to particular tastes and preferences. Thus, scientific evidence on movie portrayals of smoking points to unobserved tastes and preferences as a motivator of adolescent choices related to movie viewing. This issue deserves greater attention in longitudinal studies.

## 5. Endogenous Regressors, Sample Selection Bias, and Unobserved Heterogeneity

The analysis in this section concerns estimation and sampling issues that heretofore have not been considered by researchers that estimate longitudinal models of youth drinking and smoking. Previous articles by Geweke and Martin [131], Heckman [73], and Heckman *et al.* [132] provide insightful discussions of endogeneity and sample selection for a limited number of early smoking studies [104,107,114,118], but the analytical issues also are relevant to drinking studies and more recent smoking studies. To the best of my knowledge, the analysis in these articles has escaped the attention of public health researchers. It is useful to begin with an intuitive discussion of the problems before turning to evidence from the longitudinal studies.

Consider a sample of youth where a portion owns an alcohol-branded item (ABI). Suppose the research problem is to identify the determinates of drinking behavior, conditional on baseline ownership of an ABI (yes or no) and other covariates for demographics, attitudes toward drinking, personality traits, social environment, and so forth. The objective is to use the empirical relationship for the sample to make predictions about the effect of ABI-ownership on drinking behaviors of the adolescent population. That is, the investigator desires ultimately to predict the effect on drinking from reducing or eliminating ABIs for the population as a whole. *Endogeneity* occurs when an independent variable included in the model is potentially a choice variable or is correlated with unobservables included in the error term of the regression equation. The problem is illustrated by assuming that each youth has a baseline endowment of observable and unobservable personal and social characteristics. Observable characteristics might include age, gender, race/ethnicity, school performance, family drinking, and peer drinking. Unobservable characteristics might include genetics, risk propensity, discount rate on future health, feelings of invulnerability, curiosity about drinking, schooling prior to baseline, attitudes toward authority, crime and other deviance, family health status, family wealth, social status, legal environment, market prices, and so forth. Note that many of the unobservables may predate the baseline survey, such as genetic characteristics or early upbringing.

The dependent variable for drinking behavior and the covariate for ABI-ownership are observed for all youth in the sample. ABI-ownership is endogenous if individuals’ decision to acquire an ABI is correlated with unobservables that affect drinking behavior. For instance, if curiosity affects ownership and is an omitted covariate in the regression for drinking behavior, then the failure to control for curiosity will yield a biased estimate for ABI-ownership due to its correlation with the error term. The bias is positive in this example, so the effect of ABIs on drinking is overstated. The least-squares estimator is biased and inconsistent (asymptotically biased), so an alternative estimator is often desirable. It is a characteristic of many econometric studies that investigators attempt to model both the relationship between the “input” factors determining the outcome (drinking) and the choices leading to observation of some of those factors [132]. Examples of potentially endogenous regressors are ABI-ownership, attendance at R-rated movies, and self-reported exposure to advertising. The instrumental variable technique is often used for endogenous regressors and is discussed below.

*Selection bias* occurs when the dependent variable is only observed for a restricted, non-random sample. The problem may arise due to non-participation (“refusals”) during the baseline survey or from attrition (“drop-outs”) during the follow-up survey. At both points in time, individuals decide whether to participate or not, so there is *self-selection* in the sample used in the analysis. In his seminal article on this problem, Heckman [68] demonstrated that bias is created because “fitted regression functions confound the behavioral parameters of interest with parameters of the function determining the probability of entrance into [or exit from] the sample” (p154). Selection bias also may arise if the investigator decides to screen the data in various ways, such as pre-screening of survey participants, favoring longer time intervals over shorter intervals, or arbitrarily discarding observations with missing data. Selection by investigators also can lead to a non-random sample.

Suppose that owners and non-owners of ABIs are chosen randomly from the population and have similar endowments of observable and unobservable characteristics. A randomly-chosen baseline sample should have average characteristics for ABI-owners and non-owners that mirror the average characteristics of the population. However, participation in the surveys is a personal decision, so the respondents are unlikely to be randomly distributed. Sample selection bias arises when some component of the participation decision also is relevant to drinking outcomes, but is unobserved [133]. Accounting for observables is reasonably straight-forward, but controlling for unobservables is not. That is, when the relationship between the survey participation decision and the drinking decision is purely through observables, appropriate specification of the regression equation will account for differences among individuals in the sample. Sample selection bias will not arise solely because of differences in observable characteristics. If the participation decision also depends on unobservables (curiosity, attitudes toward authority, family wealth, *etc.*) and these characteristics are correlated with unobservables affecting respondents’ drinking decision, then the model is misspecified. If the analyst estimates a regression of drinking behavior conditional on only observables, the model fails to account for an additional process or influence on behavior, namely, the process that determines whether the respondent participates in the baseline survey or drops-out of the follow-up survey. A bias arises due to sample self-selection, which is another way of saying the sample is non-random due to behavioral choices by participants. There are several ways for dealing with selection bias such as insuring high response rates; conducting a follow-up survey; the Heckman two-stage approach; and other estimation methods [69,70,131,133]. Finally, it is clear that in longitudinal studies both endogeneity bias and sample selection bias can be present in the same study.

### 5.1. Endogeneity Bias in Longitudinal Studies

In econometrics, statistics, and epidemiology, the problem of endogeneity occurs when an explanatory variable is correlated with the error term in the regression model. This implies that regression coefficients estimated by ordinary least-squares (OLS) are biased and inconsistent. The correlation may arise due to omission of a relevant confounding variable (specification bias), measurement errors in an explanatory variable (errors-in-variable bias), or joint determination of an explanatory variable with the dependent variable (simultaneity or “reverse causality” bias). The method of instrumental variables (IV) is frequently used to deal with issues of endogeneity [64,134]. Briefly, in the IV model there are one or more variables (the “instruments”) that affect the endogenous explanatory variable, but *only* impact the dependent variable through the explanatory variable. For example, suppose that parental political beliefs are correlated with ABI-ownership by a son or daughter, but are not correlated with the youth’s drinking behavior. In order for political beliefs to be a valid instrument, the following conditions must hold: (1) the instrument must be correlated with the endogenous explanatory variable, conditional on other covariates; (2) the instrument cannot be correlated with the error term in the equation of interest, otherwise it is an “invalid” instrument; and (3) the selected instrument should be a “strong” predictor of the endogenous variable in question, otherwise the predicted values of the endogenous variable will have “too little” variation. A “weak” instrument is a variable that is weakly correlated with the endogenous explanatory variable [127,133,134]. For instance, in a study of the effects of smoking on adult physical functional status (*i.e.*, health), Leigh and Schembri [135] argue that smoking affects health (and vice versa), but cigarette prices only affect smoking. Hence, they choose price as an instrument in the IV method to obtain predicted values for smoking for inclusion in the health regression, and report that “the true effect of smoking on health is larger than conventional methods have estimated” (p290).

The standard approach to IV estimation—referred to as Two-Stage Least Squares (2SLS)—is, first, to regress the endogenous explanatory variable on the instrument(s) and all exogenous covariates (age, gender, race, school performance, *etc.*) to obtain predicted values for ABI-ownership. In many cases, the first-stage regression is of interest in its own right because it explains ownership behavior. Call the predicted values ABI-hat. Second, regress drinking behavior on ABI-hat and all other exogenous covariates, which will yield a statistically consistent estimate for ABI-ownership. Depending on the software used, the correct standard errors in the IV procedure may require additional estimation [64]. Some caution is required if the model is inherently nonlinear [136]. Various statistical tests for potential endogeneity also are available such as the widely-used test suggested by Hausman [137]. None of the longitudinal studies of youth drinking and smoking seem to have recognized the potential for endogenous regressors. In particular, all advertising and marketing covariates are treated as exogenous. Hence, all longitudinal studies are potentially subject to endogeneity bias. However, a few studies do provide descriptive analyses of the advertising covariate. Note that dealing with endogeneity also requires addressing the problem of the correct set of covariates for advertising receptivity and exposure, which is equally troublesome. I briefly summarize the results in two studies of alcohol behavior and two studies of smoking behavior that describe underlying aspects of the ownership or exposure decisions, which demonstrate non-random assignment among participants or potential endogeneity.

McClure *et al.* [85] study the effect of ABI-ownership on initiation of drinking and binge drinking in a sample of 6500 adolescents, ages 10–14 years at baseline in 2003. Follow-up surveys were conducted at 8, 16, and 24 months, and information on ABI-ownership was acquired at 8 months. As part of the study, they examine the characteristics of adolescents that own ABIs using a multivariate logistic regression and also report how the ABI was obtained. ABI-ownership is strongly and positively related to age of respondent, access to alcohol at home, sensation seeking, exposure to alcohol portrayals in movies, and susceptibility to alcohol use at baseline. Ownership also is positively related to peer drinking, rebelliousness, extracurricular activities, but not to television viewing, parental drinking, parenting style, or academic performance. Variables for blacks and Hispanics are negative predictors of ownership. All of these variables are treated as exogenous and are covariates in regressions for initiation of alcohol use and binge drinking. Because ABI-ownership is a behavioral choice, it is possible it is an endogenous regressor, which imparts bias to the reported coefficient estimates. In their discussion of the study’s limitations, the authors fail to recognize or comment on this problem. Sargent *et al.* [88] study the effect of movie-alcohol use on drinking initiation in a sample of 550 adolescents, ages 10–14 years at baseline in 1999. The follow-up survey is conducted on average at 17 months (range 12–26 months) after the baseline survey. As part of the study, they examine bivariate logistic relationships between movie-alcohol exposure (in hours) and other covariates in the drinking regression. They report that exposure to movie-alcohol use is significantly higher in older adolescents; in males; adolescents who smoke; and in those with lower parental education, poor school performance, lower levels of self-esteem, lower maternal support, and higher levels of rebelliousness and sensation seeking. All of these variables are treated as exogenous and included in a logistic regression for drinking onset. Because movie exposure is a behavioral choice, it is possible it is an endogenous regressor, which imparts bias to the reported coefficient estimates. In their discussion of the study’s limitations, the authors fail to recognize or comment on this problem. Because the ownership and exposure measures are shown to be predictable in a reasonable manner, the partial results in both drinking studies demonstrate that a multicausal model is required to understand risky behavior by adolescents [132].

Sargent *et al.* [118] study the effect of CBI-ownership on smoking status (6-point index) in a sample of 480 adolescents, ages 9–16 years at baseline in 1996. They truncate the sample and exclude established baseline smokers. Follow-up surveys are conducted in 1997 and 1998. As part of the study, they examine bivariate logistic relationships between CBI-ownership and other baseline covariates. Ownership is positively related to male gender, smoking by family and peers, and smoking status at baseline. In other words, male smokers at baseline are more likely to own or are more willing to use a CBI, regardless of other characteristics (age, school performance, parental education). No mention is made in this study of the possibility that CBI-ownership is an endogenous variable. Instead, Sargent *et al.* [118] claim that “these data provide strong evidence supporting a causal link between progression of smoking among adolescents and changes in their response to cigarette promotional items” (p326). This conclusion is premature. Wilkinson *et al.* [125] study the effect of movie-smoking exposure on smoking experimentation in a sample of 1300 Mexican-heritage adolescents, ages 11–13 years at baseline in 2001. Follow-up surveys were conducted every six months in 2002 and 2003. As part of the study, they determine participants’ level of exposure to movie smoking using 50 box office hits for the years 1999 to 2004. For each of eleven pairs of covariates, they calculate mean levels of exposure to smoking in movies and perform statistical tests for significant differences. Based on these simple comparisons, movie exposure is more prevalent for older adolescents; for males; youth born in the US; and those exhibiting higher-levels of risk-taking, anxiety, acculturation, school detention, and parental education. Movie exposure also is higher for baseline experimenters, adolescents with other smokers in the household, and those respondents who had friends who smoke. The authors fail to recognize that movie exposure also is a choice variable that might be endogenous in the smoking regression.

As noted by Geweke and Martin [131], “youth who have strongly favorable attitudes and preferences toward smoking will plausibly pay more attention to information of various kinds about cigarettes, are plausibly more likely to convey their preferences through the use of promotional products, and are plausibly more likely to start smoking at some point” (p119). In a similar vein, Heckman *et al.* [132] argue that “participants who have greater preferences for smoking might seek out and obtain more tobacco-related items, even if the items have no independent causal effect on their desire to smoke” (p43). Thus, a finding that CBIs or movie-exposure is associated with smoking behavior does not demonstrate or prove causality. These four examinations of receptivity and exposure in the smoking and drinking studies are consistent with this behavioral interpretation and inconsistent with a random assignment of these variables among adolescents. More complex and complete multicausal models of choice behavior are required if longitudinal researchers desire to better measure youth preferences for advertising and drinking or smoking outcomes.

### 5.2. Selection Bias in Longitudinal Studies

Sample selection bias arises when a rule other than random sampling is used to sample from the underlying population of interest. Distorted or unrepresentative sampling may be present in a study due to decisions by investigators or more frequently, and subtly, as a result of self-selection decisions on the part of individuals being surveyed. Adolescents (or their parents) decide whether or not to participate in a school-based survey or telephone survey. Adolescents (or their parents) decide whether to participate in one or more follow-up surveys. In his seminal paper, Heckman [68] outlined an economic model of self-selection and used it to demonstrate that selection bias can be treated as a specification error that leads to biased estimates in an OLS regression. That is, variables that affect the participation decision are correlated with unobservables that affect the outcome of interest, such as adolescents’ drinking or smoking behaviors. According to Vella [138], it may be possible to detect selection bias by studying differences in observables across subsamples. If the subsamples differ importantly, this may indicate that there are unobservables that are correlated with the observed outcome variable. Heckman’s solution for dealing with the self-selection problem is a two-stage model. In brief, it is possible to obtain an estimate of the unobservables by, first, estimating a regression for participation. That is, use the information on attrition in the follow-up sample and covariates to estimate a regression in which the binary dependent variable is the participation decision. The expected values of the errors from the first-stage regression are used to calculate a new variable (called the inverse Mills ratio). The second-stage regression is for the outcome, but with the addition of the new variable that corrects for misspecification arising from self-selection by survey participants. Because Heckman’s procedure has a rather limited structure and is highly parameterized, a number of other statistical solutions have been proposed [60,61,69,70,138,139].

Are longitudinal survey studies subject to sample selection bias? Three pieces of information can be presented that pertain to this issue. First, an examination by Geweke and Martin [131] of the CTS sample used by Pierce *et al.* [114] to study the influence of advertising receptivity on adolescent smoking. Second, the information contained in studies that estimate the outcome regression for important subsamples by age, gender, race, country of origin, and parenting style. Third, information contained in many longitudinal studies on attrition in the follow-up sample.

In their critique of longitudinal studies of adolescent smoking, Geweke and Martin [131] obtained the raw data used by Pierce *et al.* [114]. They use these data to demonstrate that Pierce’s sample is not random due to selection bias. Westat Inc., the firm that collected the CTS data on adolescent smoking, started with a random-digit dial survey for 78,000 randomly-selected telephone numbers for the entire state of California. Of this number, 44,000 were residential household telephone numbers. From the household sample, there were 10,000 refusals and 3000 other non-responses. These omitted households contained an estimated 3006 adolescents eligible for the youth part of the survey. Because Westat provided information on the broad purpose of the survey, Geweke and Martin [131] argue that “it is plausible that the 22.9% of the random sample of households that refused to complete the screening interview have attitudes and experiences relating to smoking . . . that are not representative of the population at large – that is, that are not random” (p122). In the remaining 31,000 households, there were 6862 adolescents who were eligible for the youth portion of the survey, but 727 (11%) of these individuals refused to participate and 604 were unavailable for other reasons. A total of 5,531 interviews were completed for the baseline, but 320 youth (6%) refused to participate in the follow-up survey, 371 were unavailable for other reasons, and 1464 could not be located. The final sample size was 3,376, which is only 34.2% of the randomly-selected youth at the start. This is far from the 85−90% that is the target response rate in survey studies and not even close to the 80% rate reported by Pierce *et al.* [114]. Two other CTS studies [107,111] also report low completion rates of 47**−**49%, but fail to address selection bias in these data. Geweke and Martin [131] then go on to study the probability of re-interview. Using a logistic regression, they show that re-interview was less likely for older youth, males, non-whites, less academically inclined, and smokers. They use this information to reject the hypothesis that the follow-up sample is a random subsample of the baseline sample. Finally, they study possible reassignments of the excluded respondents on Pierce’s study results. Geweke and Martin [131] observe that “even modest sample selection problems can account for the relationship between receptivity and smoking progression found in Pierce *et al.*” (p127). They conclude that this study is “so beset by sample selection problems that it cannot be used with any reliability” (p129), even for the limited purpose of descriptive-inferential modeling.

A second piece of information on selection bias is found in studies that estimate subsample regressions, which tend to demonstrate that empirical results for advertising receptivity and exposure differ importantly depending on how the data are divided. If the full sample is biased in some manner toward the subgroup with a stronger response to advertising, the study results will be biased in the positive direction. The opposite bias is equally likely, but the issue in part is the direction of bias due to self-selection at baseline, which is not observed or reported in most studies. In alcohol studies, separate results by gender are reported by Caswell *et al.* [75] for New Zealand youth. They report that liking of advertising is marginally significant for males and insignificant for females. Connolly *et al.* [78] also studied New Zealand youth using similar survey data. They find that for males and females, none of the media variables are significant for wine/spirits, but some media variables for beer are significant for males and some are negative for females. Fisher *et al.* [80] also divide the sample by gender. Ownership of an ABI is significant for both males and females and the coefficient magnitudes are similar. Snyder *et al.* [89] present results for the full sample (ages 15−26) and for a subsample of younger participants (ages 15–20). The results for three advertising variables are similar, except that advertising exposure within-individuals is not significant in the full sample. Only mean advertising exposure is marginally significant for both samples, but it is subject to measurement errors. The subsample results in some studies of adolescent drinking are suggestive of selection bias, but the evidence is limited to a few simple comparisons. As is common practice in econometrics, it would be helpful for investigators to provide additional empirical results in the form of a sensitivity analysis for important subsamples by gender, race/ethnicity, school performance, parental drinking, parenting style, and so forth.

In smoking studies, Charlton and Blair [106] report results by gender for youth in the UK, ages 12–13 years. The risk of smoking onset is somewhat greater for girls compared to boys. In particular, ability to name at least one cigarette brand is significant for girls and not for boys. However, having a favorite cigarette advertisement and watching cigarette-sponsored TV sports programs is insignificant for both genders. Jackson *et al.* [112] report separate results for black and white adolescents, ages 12−14 years. After adjusting for covariates, high-exposure to R-rated movies is insignificant for blacks and significant for whites. However, variables for hours of TV viewing per day and per week are insignificant for both races. Pierce *et al.* [115] report separate results for respondents with more- and less-authoritative parents. Never-smokers at baseline with more-authoritative parents are more likely to begin smoking if they also had a high-level of receptivity to tobacco advertising and promotion. The receptivity variables at all levels are insignificant for respondents with less-authoritative parents. These findings are somewhat surprising and Pierce *et al.* [115] interpret their results in terms of communication theory, but ignore the possibility of selection bias. Wilkinson *et al.* [125] report separate results by country of origin for a sample of Hispanic adolescents (Mexican born, US born). Exposure to smoking in movies is significant for Mexican-born youth, although the authors note that the number of high-exposure youth is small for both subsamples. Overall, the subsample results in some studies suggest that unobserved influences may create sample selection bias according to gender, race/ethnicity, curiosity, and parenting style. As a consequence, sensitivity analyses are recommended.

Finally, many longitudinal studies report information on attrition of participants in the baseline sample, *i.e.*, those respondents who dropped-out of the follow-up sample. In general, the objective of these analyses is to demonstrate that the drop-outs have characteristics that are broadly representative of the baseline sample; that is, attrition does not result in a sample that is biased toward a positive result for the advertising covariate [83,104]. As discussed above, attrition in the follow-up sample is only part of the problem as baseline refusals also are relevant. Most analyses are rather descriptive or incomplete. For example, Hanewinkel *et al.* [81] simply report that sample attrition is higher for younger participants, males, sensation-seekers, having parents who drink less frequently, less movie exposure, and other variables. They fail to provide more details regarding the causes or effects of attrition. A few studies report results from a multivariate analysis. For drinking studies, Sargent *et al.* [88] report that attrition is significantly associated with parental education, school performance, smoking status, and rebelliousness, but the overall explanatory power of the regression is poor. This may be due to randomness among drop-outs or to unobserved characteristics in the study. Stacy *et al.* [90] report the construction of a risk-based “propensity score” for drop-outs, but many details are omitted. For smoking studies, Sargent *et al.* [118] report results from an experiment with different levels of predicted smoking uptake for the 122 drop-outs in their sample. They conclude that their results are not sensitive to increased smoking propensity or higher advertising receptivity among drop-outs. Titus-Ernstoff *et al.* [124] report the results of a logistic regression for drop-out status conditional on all other baseline covariates. Dropping out of the study is associated with rebelliousness, parental smoking, and lower parental education. Non-whites are somewhat more likely to drop-out, other things being equal. Overall, the results in some attrition analyses are suggestive of selection bias according to race, parental drinking, and parental smoking, but these analyses do not include baseline refusals.

## 6. Discussion and Alternative Research Designs

A critical assessment of longitudinal studies of youth drinking and smoking reveals a number of shortcomings and omissions in the study methodologies. These problems mean that the studies do not demonstrate causality between advertising-marketing exposure and youth drinking and smoking behaviors. First, specification errors arise when an empirical model omits a relevant covariate or explanatory variable. Many model specifications are ad hoc and not guided by a well-defined theoretical model. Greater use of classifications for the explanatory variables would help, but virtually all studies omit market-area variables such as product prices and regulations. A simple approach is the inclusion of fixed-effects binary indicator variables, but this may not help if there is interest in the effect of specific regulations. Some econometric studies reviewed below have included policy indexes as an alternative to indicator variables. Formal methods for model selection are available, such as Hendry’s general-to-specific procedure [69,96]. Sensitivity analysis is required to test the robustness of empirical results, with particular attention to the range of results for the advertising and marketing variables. Of special importance is the lack of attention to models with several advertising and marketing variables. Rather than test a general model, investigators have reported separate regressions for individual marketing variables, either in the same paper or in separate papers using the same data. This biases the results and overstates any possible effect of advertising on the outcomes.

Second, measurement errors are associated with current use of advertising receptivity and exposure variables, which creates a bias due to endogeneity. In order to have policy relevance, receptivity-exposure measures should be related in some fashion to actual advertising campaigns or public policies affecting advertising and marketing. Most measures are not demonstrated in any manner to have policy implications, other than the broad policy of total bans of alcohol and tobacco advertising and promotion. Receptivity measures are rather broad and amorphous, and focused on brand identification (favorite ad, brand recall, branded merchandise). Brand loyalty is not the same as a causal effect of advertising on behaviors, but numerous studies draw this connection based on receptivity and other brand-related variables. As noted by Heckman [73], this confuses personal preferences with advertising exposure. The results are especially fragile for moderate and low levels of receptivity, but this raises an issue of measurement error or possibly omitted or mediating variables. Moderating effects of advertising also deserve greater attention than it has received, with reporting of full empirical results and standard errors. Given the results assembled to date, there is no clear evidence of a positive effect of advertising and marketing on alcohol and tobacco outcomes. The results are particularly fragile for mass-media variables, but even the results for alcohol-branded items (ABIs), cigarette-branded items (CBIs), and movie-exposure contain inconsistent results. Overall, only 21 of 63 estimates (33%) for drinking behaviors are statistically significant in Table 3. For smoking behaviors, only 31 of 63 estimates (49%) are statistically significant in Table 4. Many of the significant coefficients are for movie-exposure where model misspecification is an important issue. Results for movie-exposure must be regarded as tentative until more complete models with several advertising covariates are reported.

Third, the theoretical models that underlie longitudinal studies do not admit the possibility that advertising receptivity and exposure are determined endogenously. The result is a biased set of estimates that offer little in the way of causal relationships. Ownership of an ABI or CBI is a behavioral choice in the same manner as a youth chooses (or refuses) to drink or smoke. Hence, models of adolescent behaviors must entertain the possibility of a multicausal model, wherein the choice behaviors affecting advertising receptivity and exposure are modeled as well as the choice behavior for the outcome in question. Instead, longitudinal studies adopt a simple research design in which the advertising variable represents an exogenous “treatment” and the relationship being measured is akin to a “dose-response” relationship. The point here is that advertising receptivity and exposure are not randomly assigned across survey participants, but rather are the outcomes of choice behavior by the participants. The evidence reviewed from several studies is entirely consistent with a non-random assignment. In order to be useful for alcohol and tobacco policy, longitudinal studies must consider the use of instrumental variables in order to control for or rule out underlying preferences as the “cause” of alcohol and tobacco behaviors. As noted by Heckman *et al.* [132], longitudinal “studies do not accurately model human behavior, as these studies ignore how human choice affects the measurement of both ‘treatment’ and outcome . . . [and] not addressing the potential role individual choices have in shaping the choice or acceptance of a tobacco item or other receptivity measures, which are taken to be surrogates for advertising in many public health areas, makes cited results unreliable” (p43).

Fourth, although longitudinal studies go to great lengths to ensure random samples of participants, the studies are deficient in attending to issues of sample selection bias, especially bias introduced at the time of the initial or baseline survey. Participation in the survey is a choice-outcome by adolescents (and their parents), which under the right circumstances can be observed and modeled. The procedures used presently to check on attrition bias are too simple and inadequate to deal with the task of detecting and controlling for self selection. The review in this paper noted three pieces of information that are at least suggestive of selection bias. First, an examination by Geweke and Martin [131] of the raw CTS data used in [114] shows that there is substantial non-response at baseline as well as attrition in the follow-up sample. Possible reassignments of refusals in this study render void the conclusions. Second, a number of studies offer results for subsamples, which might reveal important differences among participants that could bias the results or have important policy implications. Results for advertising and receptivity variables are in some instances quite sensitive to modeling of the subpopulation, which is a less formal method for detecting selection bias [138,139]. Third, many longitudinal studies report information on attrition during the follow-up sample, which is generally low and aided in part by the short-duration from baseline to follow-up. This poses a dilemma for survey studies because choosing a short duration can restrict the outcome to a group of more receptive individuals who may smoke or drink for reasons that pre-date the baseline survey. Choosing a longer duration leads to greater attrition from the baseline sample. For example, in the four-year study by Audrain-McGovern *et al.* [126], the initial population of 9th graders in five northern Virginia high schools was 2,382, but the baseline sample was only 1,123 students (47%) due to refusals and nonresponses. It is unclear in many longitudinal studies whether attrition of this magnitude occurs (or matters) since the focus is usually on only the follow-up sample.

Given the important limitations of longitudinal studies, it is useful to consider alternative econometric methodologies for determining the effects of advertising and advertising regulation. Table 5 presents a selected group of nine alcohol studies and nine tobacco studies. The study lists are not comprehensive, but rather are chosen to illustrate several important analytical points regarding estimation and policy-related results. First, it is possible to model advertising expenditures or public policies that affect advertising such that the empirical results have policy implications. The studies in Table 5 include examinations of advertising expenditures, partial advertising bans, and comprehensive bans. Many of the studies illustrate quasi-experimental designs. Second, the studies demonstrate the important role that product prices have for outcomes. The strength of longitudinal studies is that they focus modeling efforts on a subpopulation of interest, namely adolescents. A major weakness of longitudinal studies is that they do not model advertising in a useful manner or account for market variables such as product prices. The studies in Table 5 include four time-series studies for Australia, Canada, and Sweden [140,141,146,150]; seven cross-sectional studies that use survey data for the Canada, Sweden, US, and multiple countries [97,98,142,144,147–149]; five panel data studies for US states and multiple OECD countries [66,143,145,151,152]; and two meta-analyses [51,153]. There are eight studies in the table that include results for youth [51,98,144,145,147–149,152]. Although two studies [145,149] report some positive results, the effects of advertising in these studies are modest or inconsistent across subpopulations [51]. In some cases, regulations have a short-run effect that dissipates with time [140,150] or there are cross-product compensating effects [143]. These important modeling considerations are omitted from longitudinal studies.

There are several conclusions to be drawn from Table 5. First, there is no evidence that higher-frequency data are important. A study using monthly advertising expenditure data fails to reveal an effect of advertising on alcohol consumption [141]. This replicates the results obtained in numerous other studies using annual and quarterly data [39,40]. Second, both partial and complete bans of advertising are ineffective in reducing alcohol or tobacco use for adult and youth populations. In Paschall *et al.* [144], a composite alcohol advertising control rating is not statistically significant at standard confidence levels and hence does not affect alcohol use by youth in 26 developed countries. In Hublet *et al.* [148], bans of advertising do not have a significant effect on smoking behaviors of youth in 29 developed countries. In Nelson [152], partial and complete bans of advertising do not have an effect on youth smoking in 42 developing countries. Similar results are found in other alcohol and tobacco studies for partial bans (billboards, window displays, campus bulletin boards, distilled spirits advertising), more complete bans (bans of broadcast advertising, bans of all media), and other regulations (warning labels). Third, product price is an important variable that significantly affects drinking and smoking outcomes. All of the econometric studies in Table 5 that include prices find significant price elasticities for alcohol and tobacco, including youth behaviors. This variable is overlooked in social learning theory and omitted in longitudinal studies. Finally, the cumulative evidence in two meta-analyses [51,153] does not support the use of advertising bans as a means to reduce alcohol or tobacco consumption. As noted, this finding applies to youth as well adults. The summary of findings in Table 5 supports other reviews of advertising for alcohol [39,40] and tobacco [154,155], which also conclude that advertising bans are ineffective.

## 7. Conclusions and Policy Implications

Longitudinal studies use a quasi-experimental research design, which implies non-randomness in the assignment of treatment and comparison groups. This design requires attention to a number of statistical and econometric problems [64]. Overall, the results in the present review fail to agree with prior results in more limited reviews for alcohol [31,48–50] and tobacco [32,42,101]. My results raise important issues regarding both internal and external validity threats to research conclusions, which are largely ignored by longitudinal researchers and public health reviewers. This review has offered a number of suggestions for improving the research methods used by longitudinal investigators. First, attention needs to be paid to selecting sets of covariates that provide an adequate test of the underlying theory, including market-wide variables. Second, several methods of advertising and promotion should be investigated using the same sample. Many studies investigate only one measure, such as branded merchandise or movie portrayals. Third, exposure to advertising and marketing needs to be treated as endogenous variables involving choices on the part of youthful respondents. Given a nonrandom assignment of advertising among respondents, the instrumental variable technique should be considered as a means of handling this problem. Fourth, selection bias is an issue in longitudinal studies since participation is a personal choice on the part of youth and their parents. Failure to deal adequately with this problem results in a nonrandom sample and biased empirical results. Overall, here are many areas where improvements are needed and much work to be done if longitudinal studies are to serve as a bias for public policy.

Advertising of alcohol and tobacco is a major preoccupation of regulators in most developed countries. This stems from social costs associated with the use of these products and concern for the health and welfare of adolescents. Youth who engage in risky behaviors may not experience immediate adverse effects of drinking and smoking, but the choices they face as adults may be adversely restricted by past adolescent behaviors and outcomes. This creates concerns by public officials and advocacy groups regarding persuasive advertisements for these products, and the information and images contained in the ads or other promotions. However, it is important to remember that advertising regulation is but one of a large number of public policies that can affect alcohol or tobacco use, including controls on purchase age, possession, physical availability, product strength, warning labels, server training, education programs, taxation, and advertising content codes. Simultaneous consideration of a menu of policies should be used to select those tools that are most effective or can be implemented at a low social cost. Given a policy menu, the important lessons from this review are that, first, studies using longitudinal surveys have not established that advertising is a causal factor for youth drinking and smoking and, second, these studies cannot be used to support recommendations for advertising and marketing bans. My analysis and conclusions support several previous surveys that examine a menu of public policies available for alcohol regulation [156,157] and tobacco regulation [158,159], and which reach a similar conclusion regarding the relative importance of advertising. In conclusion, it would be useful to refocus longitudinal studies on other policy variables that importantly affect youth alcohol and tobacco behaviors.

## Figures and Tables

**Table 1.** Trends in US Youth Drinking and Smoking Prevalence (% Use).

**Year**	**1975**	**1980**	**1985**	**1991**	**1995**	**2000**	**2005**	**2006**	**2007**	**2008**	**2009**	**Change 95–09**
**30-day alcohol use (%)**												
8th grade				25.1	24.6	22.4	17.1	17.2	15.9	15.9	14.9	−9.7
10th grade				42.8	38.8	41.0	33.2	33.8	33.4	28.8	30.4	−8.4
12th grade	68.2	72.0	65.9	54.0	51.3	50.0	47.0	45.3	44.4	43.1	43.5	−7.8
College				74.7	67.5	67.4	67.9	65.4	66.6	69.0	na	1.5
Young adult				70.6	68.1	66.8	68.6	68.7	69.5	68.9	na	0.7
**30-day cigarette use (%)**												
8th grade				14.3	19.1	14.6	9.3	8.7	7.1	6.8	6.5	−12.6
10th grade				20.8	27.9	23.9	14.9	14.5	14.0	12.3	13.1	−14.8
12th grade	36.7	30.5	30.1	28.3	33.5	31.4	23.2	21.6	21.6	20.4	20.1	−13.4
College				23.2	26.8	28.2	23.8	19.2	19.9	17.9	na	−8.9
Young adult				28.2	29.2	30.1	28.6	27.0	26.2	24.6	na	−4.6

Source: *Monitoring the Future: National Survey on Drug Use*, 1975–2009.

**Table 2.** Advertising-Promotion Variables by Study: Alcohol.

**Advertising-Promotion Variable**	**Studies (ref. no.) Using This Variable**
Watching TV (e.g., number of hours per week)	[78,79,82,87,90,91]
Watching music videos on TV or VCR	[87 (2 types),^91^,^93^]
Advertising receptivity index (ABI, favorite ad, brand)	[83]
Liking of ads, awareness of ads	[75,76,80]
Brand recognition, brand recall or favorite brand	[76,83 (2 types),^90^ (2 types)]
No. of alcohol ads recalled, exposure to alcohol ads	[78,86,89,90]
Advertising expenditures in local media market	[89]
TV alcohol ads exposure	[77,79]
Sports TV alcohol ads exposure	[77 (2 types),^90^]
Radio listening	[77]
Magazine reading, magazines with alcohol ads	[77,79]
Outdoor displays (billboards, outside store ads)	[86 (4 types)]
In-store displays	[77,79]
Concession stands at events; entertainment portrayals	[77,78,79]
Own or willing to use an alcohol-branded item	[77,80,84,85]
Movie exposure & video portrayals of alcohol	[81,82,88,92,94]

**Table 3.** Empirical Estimates in Longitudinal Studies: Alcohol.

**Study [ref. no.]**	**Drinking Onset**		**Drinking Behaviors**	
	**Marketing exposure**	**Odds ratio (95% CI)**	**Marketing exposure**	**Odds ratio (95% CI)**
Casswell *et al.* [75]			Liking ofads	1.60 (0.96, 2.70)
Collins *et al.* [77]			ESPN-TV beer ads	1.08 (0.83, 1.42)
Collins *et al.* [77]			TV sports beer ads	**1.19** (1.01, 1.40)
Collins *et al.* [77]			Other TV beer ads	1.13 (0.95, 1.34)
Collins *et al.* [77]			Magazine reading	0.96 (0.87, 1.06)
Collins *et al.* [77]			Hours radio listening	1.17 (1.00, 1.37)
Collins *et al.* [77]			Beer concessions	1.01 (0.91, 1.13)
Collins *et al.* [77]			In-store beer ads	1.03 (0.92, 1.14)
Collins *et al.* [77]			Beer merchandise	**1.76** (1.23, 2.52)
Collins *et al.* [77]			Hours TV viewing	0.86 (0.73, 1.03)
Ellickson *et al.* [79]	TV beer ads	1.05 (0.64, 1.70)		
Ellickson *et al.* [79]	Magazines with ads	1.12 (0.94, 1.30)		
Ellickson *et al.* [79]	Beer concessions	1.06 (0.83, 1.40)		
Ellickson *et al.* [79]	In-store displays	**1.42** (1.10, 1.80)		
Ellickson *et al.* [79]	Weekly TV viewing	0.78 (0.69, 0.88)		
Fisher *et al.* [80]	Boys-alcohol merchandise	**1.78** (1.36, 2.33)	Boys-alcohol merchandise	0.87 (0.51, 1.48)
Fisher *et al.* [80]	Boys-awareness of ads	1.27 (0.98, 1.64)	Boys-awareness of ads	0.98 (0.58, 1.66)
Fisher *et al.* [80]	Girls-alcohol merchandise	**1.74** (1.37, 2.19)	Girls - alcohol merchandise	**1.79** (1.16, 2.77)
Fisher *et al.* [80]	Girls-awareness of ads	1.04 (0.84, 1.29)	Girls-awareness of ads	1.16 (0.77, 1.74)
Hanewinkel *et al.* [81]			Parents don’t limit movies	**2.53** (1.55, 4.12)
Hanewinkel & Sargent [82]			Hours of movie alcohol use	1.44 (0.96, 2.17)
Hanewinkel & Sargent [82]	Hours of movie alcohol use	**1.42** (1.16, 1.75)	Hours of movie alcohol use	**1.95** (1.27, 3.00)
Hanewinkel & Sargent [82]	Hours TV viewing	0.99 (0.75, 1.31)	Hours TV viewing	0.76 (0.48, 1.19)
Henriksen *et al.* [83]	Beer brand recognition	1.07 (0.93, 1.23)	Beer brand recognition	1.13 (0.93, 1.38)
Henriksen *et al.* [83]	Beer brand recall	1.10 (0.97, 1.25)	Beer brand recall	1.11 (0.94, 1.33)
Henriksen *et al.* [83]	Receptivity: moderate	1.20 (0.75, 1.90)	Receptivity: moderate	1.19 (0.62, 2.26)
Henriksen *et al.* [83]	Receptivity: high	**1.68** (1.20, 2.35)	Receptivity: high	**1.62** (1.01, 2.60)
McClure *et al.* [84]	Alcohol merchandise	**1.50** (1.10, 2.00)		
McClure *et al.* [85]	Alcohol merchandise	1.41 (0.98, 2.01)	Alcohol merchandise	**1.80** (1.28, 2.54)
McClure *et al.* [85]	Alcohol merchandise	1.57 (0.99, 2.50)	Alcohol merchandise	1.44 (0.90, 2.31)
Robinson *et al.* [87]	TV viewing	**1.09** (1.01, 1.18)	Hours TV viewing	1.01 (0.93, 1.11)
Robinson *et al.* [87]	Music TV videos	**1.31** (1.17, 1.47)	Music TV videos	1.05 (0.95, 1.17)
Robinson *et al.* [87]	VCR videos	0.89 (0.79, 0.99)	VCR videos	0.97 (0.86, 1.10)
Robinson *et al.* [87]	Computer games	0.94 (0.84, 1.05)	Computer games	1.00 (0.89, 1.12)
Sargent *et al.* [88]	Hours of movie alcohol use	**1.15** (1.06, 1.25)		
Stacy *et al.* [90]			TV ads: beer	**1.44** (1.27, 1.61)
Stacy *et al.* [90]			TV sports ads: beer	**1.20** (1.05, 1.37)
Stacy *et al.* [90]			Ad exposure: beer	**1.21** (1.04, 1.41)
Stacy *et al.* [90]			Brand recall: beer	1.17 (0.97, 1.38)
Stacy *et al.* [90]			TV ads: wine/liquor	**1.34** (1.17, 1.52)
Stacy *et al.* [90]			TV sports ads: wine/liquor	1.00 (0.88, 1.15)
Stacy *et al.* [90]			Ad exposure: wine/liquor	1.18 (0.98, 1.32)
Stacy *et al.* [90]			Brand recall: wine/liquor	1.07 (0.91, 1.26)
Stacy *et al.* [90]			TV ads: 3-drink episodes	**1.26** (1.08, 1.48)
Stacy *et al.* [90]			TV sports ads: 3-drink episodes	1.07 (0.91, 1.26)
Stacy *et al.* [90]			Ad exposure: 3-drink episodes	1.06 (0.89, 1.27)
Stacy *et al.* [90]			Brand recall: 3-drink episodes	1.17 (0.91, 1.44)

Notes: Bold entries are statistically significant; 95% confidence interval (CI) in parentheses. Table values for Ellickson *et al.* [79] are based on data and information provided by Phyllis Erickson. Additional calculations computed using Comprehensive Meta Analysis v2.2; see [51] for details.

**Table 4.** Empirical Estimates in Longitudinal Studies: Tobacco.

**Study [ref. no.]**	**Smoking Outcome**	**Marketing Measure**	**Odds Ratio (95% CI)**
Biener & Siegel [104]	Regular smoker (100+ smokes in lifetime)	Receptivity: moderate	0.98 (0.53, 1.83)
Biener & Siegel [104]	Regular smoker (100+ smokes in lifetime)	Receptivity: high	**2.70** (1.24, 5.85)
Choi *et al.* [107]	Regular smoker (100+ smokes in lifetime)	Receptivity: moderate	1.23 (0.81, 1.88)
Choi *et al.* [107]	Regular smoker (100+ smokes in lifetime)	Receptivity: high	**1.71** (1.11, 2.61)
Gilpin *et al.* [111], 1993–1999 cohort	Regular smoker (100+ smokes in lifetime)	Receptivity: moderate	**1.46** (1.10, 1.94)
Gilpin *et al.* [111], 1993–1999 cohort	Regular smoker (100+ smokes in lifetime)	Receptivity: high	**1.84** (1.15, 2.94)
Gilpin *et al.* [111], 1996–2002 cohort	Regular smoker (100+ smokes in lifetime)	Receptivity: moderate	**1.46** (1.02, 2.07)
Gilpin *et al.* [111], 1996–2002 cohort	Regular smoker (100+ smokes in lifetime)	Receptivity: high	**1.84** (1.28, 2.63)
Lopez *et al.* [113], 18 month follow-up	Regular smoker (at least one per week)	No. of brands identified on billboards	**1.15** (1.02, 1.30)
Thrasher *et al.* [123]	Current smoker (past 30 days)	Movie exposure: low	1.22 (0.59, 2.51)
Thrasher *et al.* [123]	Current smoker (past 30 days)	Movie exposure: moderate	**2.44** (1.31, 4.55)
Thrasher *et al.* [123]	Current smoker (past 30 days)	Movie exposure: high	**2.23** (1.19, 4.17)
Thrasher *et al.* [123]	Current smoker (past 30 days)	Owns CBI	1.43 (0.66, 3.11)
Dalton *et al.* [108]	Onset of smoking (any amount)	Movie exposure: low	**2.02** (1.27-3.20)
Dalton *et al.* [108]	Onset of smoking (any amount)	Movie exposure: moderate	**2.16** (1.38, 3.40)
Dalton *et al.* [108]	Onset of smoking (any amount)	Movie exposure: high	**2.71** (1.73, 4.25)
Distefan *et al.* [109]	Onset of smoking (any amount)	Receptivity: low	1.17 (0.69, 2.00)
Distefan *et al.* [109]	Onset of smoking (any amount)	Receptivity: moderate	1.34 (0.76, 2.35)
Distefan *et al.* [109]	Onset of smoking (any amount)	Receptivity: high	**1.99** (1.07, 3.72)
Gidwani *et al.* [110]	Onset of smoking (any last 3 months)	TV-viewing hours per day: low (2–3 hours)	2.00 (0.37, 10.63)
Gidwani *et al.* [110]	Onset of smoking (any last 3 months)	TV-viewing hours per day: moderate (3–4 hours)	3.15 (0.64, 15.40)
Gidwani *et al.* [110]	Onset of smoking (any last 3 months)	TV-viewing hours per day: high (4–5 hours)	**5.24** (1.19, 23.10)
Gidwani *et al.* [110]	Onset of smoking (any last 3 months)	TV-viewing hours per day: very high (5+ hours)	**5.99** (1.39, 25.71)
Hanewinkel *et al.* [81]	Onset of smoking (any amount)	FSK-16 Movie exposure: once in a while	1.19 (0.85, 1.67)
Hanewinkel *et al.* [81]	Onset of smoking (any amount)	FSK-16 Movie exposure: sometimes	**1.71** (1.33, 2.20)
Hanewinkel *et al.* [81]	Onset of smoking (any amount)	FSK-16 Movie exposure: all the time	**1.85** (1.27, 2.69)
Hanewinkel & Sargent [122]	Onset of smoking (any amount)	Movie exposure: low	**1.37** (1.09, 1.68)
Hanewinkel & Sargent [122]	Onset of smoking (any amount)	Movie exposure: moderate	**1.78** (1.39, 2.29)
Hanewinkel & Sargent [122]	Onset of smoking (any amount)	Movie exposure: high	**1.96** (1.55, 2.47)
Hanewinkel & Sargent [122]	Onset of smoking (any amount)	Favorite ad	**1.38** (1.15, 1.65)
Jackson *et al.* [112], white adolescents	Onset of smoking (any amount)	R-rated movie exposure: moderate	1.57 (0.73, 3.35)
Jackson *et al.* [112], white adolescents	Onset of smoking (any amount)	R-rated movie exposure: high	**2.67** (1.07, 6.55
Jackson *et al.* [112, white adolescents	Onset of smoking (any amount)	TV viewing hours per day: above median (>4.7)	1.32 (0.69, 2.53)
Jackson *et al.* [112], white adolescents	Onset of smoking (any amount)	TV-viewing per week: daily	1.34 (0.54, 3.29)
Jackson *et al.* [112], black adolescents	Onset of smoking (any amount)	R-rated movie exposure: moderate	0.97 (0.42, 2.12)
Jackson *et al.* [112], black adolescents	Onset of smoking (any amount)	R-rated movie exposure: high	1.75 (0.66, 4.62)
Jackson *et al.* [112], black adolescents	Onset of smoking (any amount)	TV-viewing hours per day: above median (>4.7)	0.96 (0.45, 2.01)
Jackson *et al.* [112], black adolescents	Onset of smoking (any amount)	TV-viewing per week: daily	1.15 (0.39, 3.43)
Pierce *et al.* [115], more authoritative parents	Onset of smoking (any amount)	Receptivity: low	1.76 (0.65, 4.80)
Pierce *et al.* [115], more authoritative parents	Onset of smoking (any amount)	Receptivity: moderate	2.32 (0.90, 5.98)
Pierce *et al.* [115], more authoritative parents	Onset of smoking (any amount)	Receptivity: high	**3.52** (1.10, 11.23)
Pierce *et al.* [115], less authoritative parents	Onset of smoking (any amount)	Receptivity: low	1.15 (0.38, 3.46)
Pierce *et al.* [115], less authoritative parents	Onset of smoking (any amount)	Receptivity: moderate	1.16 (0.40, 3.39)
Pierce *et al.* [115], less authoritative parents	Onset of smoking (any amount)	Receptivity: high	1.38 (0.43, 4.46)
Thrasher *et al.* [123]	Onset of smoking (any amount)	Movie exposure: low	1.01 (0.64, 1.60)
Thrasher *et al.* [123]	Onset of smoking (any amount)	Movie exposure: moderate	**1.54** (1.01, 2.64)
Thrasher *et al.* [123]	Onset of smoking (any amount)	Movie exposure: high	1.41 (0.95, 2.10)
Thrasher *et al.* [123]	Onset of smoking (any amount)	Owns CBI	1.58 (0.90, 2.76)
Titus-Ernstoff *et al.* [124]	Onset of smoking (any amount)	Movie exposure (baseline)	**1.09** (1.03, 1.15)
Pierce *et al.* [116]	Smoking experimentation	Receptivity: low	1.23 (0.75, 2.04)
Pierce *et al.* [116]	Smoking experimentation	Receptivity: moderate	1.40 (0.82, 2.42)
Pierce *et al.* [116]	Smoking experimentation	Receptivity: high	1.88 (0.99, 3.56)
Wilkinson *et al.* [125]	Smoking experimentation – Mexican born	Movie exposure (no. depictions)	**1.52** (1.14, 2.05
Wilkinson *et al.* [125]	Smoking experimentation – US born	Movie exposure (no. depictions)	1.04 (0.86, 1.27)
Pierce *et al.* [116]	Susceptible to smoking (susceptible + experimenter)	Receptivity: low	0.80 (0.46, 1.41)
Pierce *et al.* [116]	Smoking susceptibility (susceptible + experimenter)	Receptivity: moderate	1.27 (0.71, 2.28)
Pierce *et al.* [116]	Smoking susceptibility (susceptible + experimenter)	Receptivity: high	1.38 (0.70, 2.91)
Pierce *et al.* [114]	Susceptible to smoking (susceptible + experimenter)	Receptivity: low	1.32 (0.73, 2.41)
Pierce *et al.* [114]	Susceptible to smoking (susceptible + experimenter)	Receptivity: moderate	**1.82** (1.04, 3.20)
Pierce *et al.* [114]	Susceptible to smoking (susceptible + experimenter)	Receptivity: high	**2.89** (1.47, 5.68)
Weiss *et al.* [119]	Smoking susceptibility (susceptible + smokers)	Exposure to pro-tobacco media (either TV or store)	**1.89** (1.23, 2.91)
Weiss *et al.* [119]	Smoking susceptibility (susceptible + smokers)	Exposure to pro-tobacco media (TV & store displays)	**3.33** (2.16, 5.16)
Sargent *et al.* [118]	Smoking status index (0–5 scale)	Own or willing to own CBI	**1.90** (1.30, 2.90)

**Table 5.** Summary of Other Methodologies & Advertising Findings.

**Study [ref. no.]; Sample; Outcome; Methods**	**Innovations & Refinements**	**Study Findings & Conclusions**
**Alcohol advertising studies**		
Calfee & Scheraga [140]; annual time-series data for FR, DE, NL & SE; per capita alcohol use; linear & log regressions for each country.	For Sweden, alcohol advertising has been prohibited since 1979. Models include country prices, income & advertising expenditures.	Advertising coefficients are not significant for any country. The results for Sweden are not different than the other 3 countries, despite the advertising ban. Price is significant for Sweden.
Lariviere *et al.* [141]; monthly time-series data for Ontario, CN, for 1979–1987 for beer, wine, spirits & soft drinks; demand system model.	Monthly advertising expenditures for four beverages that capture “pulsing” effects across markets; advertising for four beverages, prices, income & demographics.	Advertising for beer & spirits are not significant. Negative sign for wine advertising & positive sign for soft drinks. Study concludes that “advertising is not effective in enlarging markets,” but rather promotes brand-switching.
Markowitz & Grossman [97]; 1976 Physical Violence in American Families survey; overall & severe domestic violence; probit model.	State alcohol tax, availability, illegal drug prices, restrictions on billboard advertising, restrictions on window displays & price advertising.	Restrictions on advertising are ineffective in reducing violence. Violence toward children reduced by higher alcohol taxes.
Markowitz & Grossman [142]; 1976 & 1985 Physical Violence in American Families surveys; physical child abuse by gender; probit model.	State alcohol tax, availability, illegal drug prices, restrictions on billboard advertising, restrictions on window displays & price advertising. State binary variables in some models.	Restrictions on advertising are ineffective for both genders. For females, violence toward children reduced by higher alcohol taxes in 1976 & 1985.
Nelson [143]; state panel data for 1982–1997; per capita pure alcohol use by beverage; panel data model with regional fixed effects & simulations.	Bans of billboard advertising, bans of price advertising & state monopoly control of retail stores. Study considers substitution among beverages due to regulations.	Bans of advertising do not reduce total alcohol consumption, reflecting in part substitution among beverages. Income is always significant and price is generally significant.
Nelson [66]; international panel of 17 OECD countries for 1975–2000; per capita consumption of pure alcohol; panel data model for log levels & growth rates, IV model.	Spirits broadcast advertising bans & bans of broadcast advertising for all beverages, alcohol-control policy index & drinking sentiment. Study adjusts for non-stationary data & endogeneity of the alcohol policy index.	Bans of advertising do not reduce alcohol consumption, regardless of severity. Other alcohol policies and prices have a negative effect on consumption.
Nelson [51]; meta-analysis of 21 longitudinal and panel data studies of alcohol advertising & youth drinking; meta-regression analysis.	Paper examines 23 effect-size estimates for drinking onset & 40 estimates for other drinking behaviors. Meta-regressions account for primary study heterogeneity, heteroskedasticity, omitted variables, publication quality & truncated samples.	Meta-regression results are consistent with publication bias, omitted variable bias in some studies & lack of a genuine effect for advertising, especially mass media. The paper also discusses “dissemination bias” in the use of research results by investigators & health policy interest groups.
Paschall *et al.* [144]; 2003 ESPAD Alcohol Survey for 26 countries, youth 15–17 years; current drinking & binge drinking; separate trivariate regressions.	Overall alcohol-policy index score, alcohol availability, advertising control rating & country per capita consumption. No other controls for prices, income, drinking sentiment, *etc.*	Alcohol advertising control rating is not statistically significant at standard 95% confidence level, after controlling for per capita consumption. Policy index is insignificant, but availability rating is significant for current drinking & binge drinking.
Saffer and Dave [145]; 75 media markets, US, 1996–1998 & 1997–1998, youth ages 12–17 & 12–16; past year drinking, past month, binge drinking; probit & OLS regressions.	Composite measure of local advertising expenditures. Significant in 10 of 15 cases for MTF data. Significant in 5 of 6 cases for NLSY data. Log of advertising is significant in 1 of 2 cases for NLSY. T-statistics are 2.3 or less in 14 of 23 cases.	Null effect of advertising on three MTF drinking measures for blacks. Null results for males for MTF for past month & binge drinking. Null results for NLYS for two log models. Concludes that “reduction of advertising can produce a modest decline in adolescent alcohol consumption.”
**Tobacco advertising studies**		
Bardsley & Olekalns [146]; 1962–1996 time-series data for AU; per capita tobacco consumption; rational addiction model & dynamic simulations.	Aggregate consumption in Australia peaked in the late 1960s. Real ad expenditures per capita declined after a peak in late-1960s. Most tobacco advertising banned in 1992.	Effect of pro-smoking advertising & policy interventions are small relative to economic variables for taxes, income & demographics. Evidence of forward-looking behavior; virtually all reductions in smoking due to tax increases.
Czart *et al.* [98]; 1997 Harvard Alcohol Study survey, students at 140 US colleges; current smoking & ave. daily number; probit & logistic models.	State, local and school variables for smoking policies, availability & school-level advertising bans (newspapers, bulletin boards).	Bans of cigarette advertising on campus and bans of sales of cigarettes on campus have no significant effect on smoking behavior. Price is significant for smoking participation & level of smoking.
Hammar & Martinsson [147]; 2000 county-based survey in northern SE; smoking initiation age (9–25 years); duration analysis.	Anti-smoking policies enacted in Sweden from 1955 to 1986, including 1979 laws on marketing.	Public policies do not show a significant effect on the age of smoking initiation. Age of initiation depends on gender, parental smoking & time trend.
Hublet *et al.* [148]; 2006 Health Behaviour (HBSC) survey for 29 European countries, youth 11–15 years; regular smoking by gender; multilevel model.	Country-level variables for price, public bans, advertising bans, sales to minors, vending machines, adult smoking, affluence, *etc.*	Bans of advertising & public smoking bans are insignificant. For regular smoking, price is significant for boys, but not for girls.
Lewit *et al.* [149]; 1990 & 1992 surveys of 9th grade students in 21 CN & US cities; current smoking & smoking intentions by gender; logistic model.	Site-specific smoking control variables. Includes prices, minimum age, access to vending machines, and anti- & pro-smoking media exposure. Media exposure is self-reported index for 5 media for pro-smoking & 10 media for anti-smoking.	For current smoking, pro-tobacco media significant for boys, but not for girls. For smoking intentions, pro-tobacco not significant for either gender. Concludes that “only very modest support to the notion that media-focused policy interventions will be effective.” Price significant for boys’ current smoking & girls’ intentions.
McLeod [150]; 1953–1983 time-series data for AU; tobacco & cigarette consumption; double-log model with intervention binaries.	Australia banned cigarette & tobacco broadcast advertising in 1976.	Ban of broadcast advertising has a short-run effect on tobacco use, but no effect on cigarette use. Price is significant, but income is insignificant.
Nelson [151]; international panel model for 20 OECD countries for 1970–1995; per capita cigarette & tobacco use for levels & growth rates; OLS panel model with time & country fixed-effects, IV model.	Strong bans (print + all broadcast), moderate bans (3–4 media), weak bans (TV-radio only), no. of banned media & warning labels. Study adjusts for endogeneity of advertising bans, non-stationary data & structural change.	Bans of advertising have no effect on cigarette consumption, regardless of the time period considered or the severity of the bans. Price & income are significant, but evidence of structural change beginning around 1985.
Nelson [152]; Global Youth Tobacco Survey for 42 developing countries for 1999–2001, youth 13–15 years; current smoking & ever smoked prevalence; linear probability models by gender & combined with interaction terms.	Countries with complete bans (all major media), moderate (TV or other media) bans & no media banned; warning labels & minor sales prohibited. Other covariates for availability, education, peer smoking, income, Muslin faith, former Soviet-bloc countries, *etc.*	Bans of advertising have no effect on youth smoking prevalence in developing countries for either gender or combined. Higher income levels *reduce* smoking in developing countries & smoking by peers is important. Youth in Muslin countries have lower predicted prevalence & Soviet-block countries have higher prevalence.
Nelson [153]; meta-analysis of 33 advertising elasticities for US and 16 elasticities for other countries; 19 studies of four major regulatory effects; meta-regressions.	Study adjusts for heterogeneity of estimates, heteroskedasticity & non-independence of observations. The study also reviews 50 years of advertising regulation by the FTC.	Advertising elasticities are very small and not statistically significant regardless of the time period. The 1971 ban of broadcast advertising did not affect cigarette consumption.

## Appendices: Alcohol and Tobacco Longitudinal Studies

**Appendix A.** Summary of Alcohol Longitudinal Studies.

**Study [ref. no.], location, survey dates, ages, completion %**	**Outcome measures & empirical model**	**Advertising-promotion measures & selective results**	**Covariates in final model**
Casswell *et al.* [75], Dunedin, NZ, 1990, 1993 & 1996, 18–26 years, 87%.	Wt. ave. amount per occasion; frequency of drinking for males & females. Logistic regression.	Participants at age 18 asked to rate how much they liked alcohol ads. Liking of ads is not a significant predictor for males or females.	Gender, ease of access to alcohol, access to licensed premises, living arrangement, parental consumption (at age 9), level of education, age at onset for regular drinking.
Casswell & Zhang [76], Dunedin, NZ, 1990/1991 & 1993/1994, 18–21 yesrs, 68%.	Ave. amt. of beer consumed at age 21. Structural equation model.	Liking of ads at age 18 (3-item index). Liking has effect on beer use. Brand allegiance at age 18 has effect on beer use at age 21 Null effect of liking of ads at age 18 on drinking at age 18.	Gender only.
Collins *et al.* [77], South Dakota US, 2001 & 2002, 11–13 years, 87%.	Grade-7 beer drinking (past yr.). Logistic regression.	ESPN TV beer ads, other sports TV ads, other TV beer ads, magazine reading, radio listening, concessions, in-store beer displays & beer ABIs. Significant results for only sports TV ads, radio listening & ABIs. Null effects for six other media variables.	Gender, race, adult drinking, peer drinking, parental approval, friend approval, low parental monitoring, low school grades, depressed mood, deviance, impulsivity, low religiosity, sports activity, parental education, weekly TV viewing. Some results conflict with Ellickson *et al.* [79].
Connolly *et al.* [78], Dunedin, NZ, 1985, 1987 & 1990, 13–18 years, na.	Ave. amount per occasion; max. amount; frequency of beer drinks at age 18 by males & females; separate analysis for beer & wine/spirits. Linear regression.	No. of ads recalled at ages 13 & 15; no. of moderation messages recalled at ages 13 & 15; no. of portrayals recalled at ages 13 & 15; no. of commercials recalled at age 15; ave. no. of hours of TV watched per week. For beer, null results obtained in 21 of 24 cases for ads, 24 for portrayals & 24 for moderation messages. No. of hours. of TV watched is significant for average amount consumed.	Gender, peer approval of drinking, socio-economic status, living situation, occupation.
Dal Cin *et al.* [94], nationwide US, 2003 & 8, 16, & 24 month follow-ups, 10–14 years, 66%.	Alcohol consumption in past month (derived from quantity & frequency). Structural equation model.	Movie alcohol exposure in seconds at T1 and T2 (based on survey responses for 50 movies). Movie exposure affects T3 use. Movie exposure affects T3 prototypes, expectancies & norms. Willingness to use at T3 affects T4 consumption. *Reports table of correlations.*	Age, gender, race, parental education, household income, parenting style, self-esteem, rebelliousness, sensation seeking, self-regulation, parental drinking, peer drinking, religious attendance, general media exposure, TV watched.
Ellickson *et al.* [79], South Dakota US, 1997 & 2000, 12–15 years, 82%.	Grade-9 drinking onset (past yr.) by grade-6 non-drinkers; grade-9 drinking frequency (past yr.) by grade 6 drinkers. Logistic regression.	TV beer ads, magazines with alcohol ads, beer concession stands & in-store displays. Ad variables obtained at grade 8. For onset, significant result for only in-store displays. For drink frequency, significant results for magazines & concession stands. Null results for seven other media variable.	Gender, race, adult drinking, adult approval of drinking, peer drinking, peer approval, poor grades, low parental monitoring, low religiosity, deviance, impulsivity, playing sports, alcohol beliefs, other TV viewing habits. Some results conflict with Collins *et al.* [77] for the same data set.
Fisher *et al.* [80], nationwide US, 1996 & 1998/1999, 11–18 years, 70%.	Drinking onset at follow-up by baseline nondrinkers by gender; binge drinking by baseline nondrinkers. Logistic regression.	Talked with friends about alcohol ads & ownership of ABI. For onset, significant results for ABI for boys & girls. For binge drinking, significant results for ABI for girls only. Null results for awareness for both outcomes.	Age, gender, parental drinking, sibling drinking, peer drinking, dinner at home, family composition, social self-esteem, athletic self-esteem, global self-esteem, scholastic self-esteem, smoking, expectancy score. Interaction variables with age, *etc.*
Hanewinkel *et al.* [81], Schleswig-Holstein DE, 2005 & 2006, 10–16 years, 80%.	Binge drinking onset at follow-up by baseline non-drinkers (age 15 & younger). Generalized logistic (log link) regression & path analysis model.	Frequency of exposure to movies or videos that are rated as appropriate for ages 16 and older (FSK-16 rating). Significant results for three levels of viewing FSK-16 movies (once in a while, sometimes, all the time). *Reports determinates of exposure.*	Age, gender, parental drinking, peer drinking, parenting style, school type, school performance, sensation seeking, rebelliousness.
Hanewinkel & Sargent [82], Schleswig-Holstein DE, 2005 & 2006, 10–16 years, 79%.	Alcohol use outside of family context; binge drinking. Generalized logistic (log link) regression.	Alcohol use in movies (respondent’s imputed exposure in 50 randomly selected movies); TV watching time. Significant results for hours. of movie exposure. Null results for TV watching time for both outcomes. *Reports cross-tabulation for exposure.*	Age, gender, parental drinking, peer drinking, parenting style, school type, school performance, sensation seeking, TV in bedroom.
Henriksen *et al.* [83], Tracy, CA US, 2003 & 2004, 11–13 years, 71%. *Attrition analysis.*	Onset of alcohol use by baseline nondrinkers (6–8th grades); current drinker (at least 1–2 days in past month). Logistic regression.	Alcohol marketing receptivity index (owned promo item, brand name of favorite alcohol ad); brand recall & brand recognition. For onset & current drinking, significant results for high receptivity. Null results for brand recognition, brand recall & moderate receptivity. *Reports cross-tabulation for receptivity.*	Grade level, gender, race, peer drinking, peer perceived prevalence, peer perceived approval, school performance, supervision after school, risk taking (3-item index).
McClure *et al.* [84], New Hampshire & Vermont US, 1999 & 2000/2001, 10–14 years, 67%.	Drinking onset by baseline nondrinkers (5–8th grade). Logistic regression.	Ownership of ABI (determined at follow-up). Ownership of ABI is significantly related to drinking onset, but it is the only advertising-marketing covariate. *Reports cross-tabulation for ABIs.*	Grade level, gender, peer drinking, parental education, parenting style, ever tried smoking, rebelliousness, sensation seeking.
McClure *et al.* [85], nationwide US, 2003, 2004 & 2005, 10–14 years, 74%.	Drinking onset; transition to binge drinking at 8-16 month follow-ups & 16–24 month follow-ups. Logistic regression.	Ownership of ABIs assessed at 8, 16 & 24 months. Exposure to alcohol in movies & TV viewing are unreported covariates. Mixed results for ABIs. *Reports determinants of ABIs.*	Age, gender, parental drinking, peer drinking, parental education, income, parenting style, alcohol access at home, school performance, extracurricular activities, sensation seeking, rebelliousness.
Pasch *et al.* [86], Chicago, IL US, 2003 & 2005, 11–12 years, 63%.	Alcohol behavior index (5-item index) at grade-8 for baseline nondrinkers & baseline drinkers. Mixed-effects regression.	Outdoor ads index (billboards, outside stories); outdoor brand-only ads; outdoor youth-oriented ads; index of exposure to alcohol ads in six other media (inside stores, community events, magazines, TV, radio, internet). Null results for alcohol behavior for three outdoor ad measures for baseline nonusers and users.	Baseline value of outcome, school socioeconomic status, exposure to other forms of alcohol ads, awareness of outdoor ads. Age and gender interaction terms are insignificant & are excluded in final model.
Robinson *et al.* [87], San Jose, CA US, 1994 & 1996, 14–15 years, 55%.	Onset of drinking by baseline nondrinkers; drinking maintenance by baseline drinkers (9th grade). Logistic regression.	Hours. spent watching TV & hours spent watching music videos. Both variables are significant for onset of drinking, but not for maintenance. Null results for computer-video games for onset & 4 media for maintenance.	Age, gender, race, hours. of other media use (computer, other videos).
Sargent *et al.* [88], New Hampshire & Vermont US, 1999 & 2000/2001, 10–14 years, 67%.	Drinking onset by baseline nondrinkers. Logistic regression. *Attrition analysis.*	Alcohol use in movies (reported exposure in a set of 50 movie films); significant effect of exposure on drinking onset (only media variable). *Reports determinates of exposure.*	Grade level, gender, parental education, maternal support, maternal control, school performance, self-esteem, rebelliousness, sensation seeking, ever smoked a cigarette.
Snyder *et al.* [89], 24 media markets US, 1999–2001 (in 4 waves), 15–26 years & 15–20 years, 31%.	No. of drinks consume in past month (T4), conditional on ad exposure at T1. Multilevel Poisson regression with a log-link function.	Ad exposure index from 2 questions for each of 4 media (TV, radio, magazines, billboards); industry ave. measure of alcohol ads in local market for 4 media (TV, radio, newspapers, outdoor) in 1999/2000, deflated by population size only. For 15–20 year olds, small effect of market-level advertising and mean advertising exposure, but not sales per capita.	Age, gender, race, education level, baseline drinking, market alcohol sales per capita, time. Interactions of ads exposure with age and time.
Stacy *et al.* [90], Los Angeles, CA US, 2000 & 2001, 12–13 years, 75%. *Attrition analysis.*	Alcohol use in grade 8 by beverage (beer & wine/spirits); 3-drink episodes. Logistic regression.	Three indices for TV alcohol ad exposure; 2 memory tests for ads recall & brand recognition. Watched TV index is significant for beer use, wine/liquor use & 3-drink episodes. Watched TV sports index and self-reported TV alcohol ads index are significant for beer use only. No significant results for 2 memory tests. *Reports correlation with covariates.*	Gender, race, adult drinking, peer drinking, drinking norms, intentions to drink, prior beer use, prior wine/spirits use, sports participation, general TV viewing. Unclear which of these variables are in the final model. Interactions with gender, race, and prior alcohol use are insignificant.
Van den Bulck & Beullens [91], Flanders BE, 2003 & 2004, 13–16 years, 65%.	No. of drinks while going out (to a bar, disco, *etc.*) on a scale from 0 to 9+ drinks. Linear regression.	Baseline hours of TV viewing per day; frequency of music video viewing. Significant results for both variables, but unclear if these measure exposure to alcohol ads. *Reports cross-tabulations by gender.*	School year, gender, pubertal status, baseline drinking status, smoking status.
Wills *et al.* [92], nationwide US, 2003, 2004 & 2005 (4 waves), 10–14 years, 70%. *Attrition analysis.*	Drinking onset index at T2 & T3; binge drinking at T2 & T3. Structural equation model.	Alcohol use in movies (in a set of 50 movies at T1, T2 & T3). Statistically significant result for direct effect of movie exposure at T1 on alcohol use index. Null result for direct effect of T2 movie exposure on T2 alcohol use. *Reports table of correlations.*	Age, gender, race, family structure, school performance, parental drinking, peer drinking, mother’s responsiveness, rebelliousness, sensation seeking, self-control, alcohol availability, alcohol expectancy.
Wingood *et al.* [93], nonurban US, 1996–1999, 14–18 years, 92%.	Alcohol use at 12-month follow-up. Appears to combine baseline drinkers and nondrinkers. Logistic regression.	Self-reported no. of hours. of exposure to rap music videos at baseline. Significant effect of rap music videos on onset of drinking, but covariates unclear. *Reports cross-tabulation.*	Age, parents’ monitoring, family composition, family’s public assistance, employment status, extracurricular activities, religious participation, HIV intervention, baseline alcohol use. Final model is unclear.

**Appendix B.** Summary of Tobacco Longitudinal Studies.

**Study [ref. no.], location, survey dates, ages, completion %**	**Outcome measures & empirical model**	**Advertising-promotion measures & selective results**	**Covariates in final model**
Alexander *et al.* [102], New South Wales AU, 1979 & 1980, 10–12 years, 87%.	Change in smoking status from baseline (onset, quit, continued, nonsmoker). Logistic regression, but log-odds ratios not reported.	Approval of cigarette ads at baseline. Onset (adoption) of smoking is positively related to approval of advertising. Quitting is negatively related to approval. Smoking education classes are marginally related to onset, but not to quitting.	Age, parental smoking, sibling smoking, peer smoking, weekly spending money, teacher’s smoking, teacher’s gender, urban location, private school, alcohol use, smoking education classes. Interactions.
Armstrong *et al.* [103], AU schools, 1981 & 1982/83, 11–13 years, 82% & 64%.	Change in smoking status in prior 12 months (onset, continued) by gender. Stepwise logistic regression, but log-odds ratios not reported.	Perceived attraction to cigarette ads at baseline. For boys and girls, advertising is unrelated to onset at one-year follow-up and positively related at two-year follow-up. Smoking education classes have a significant negative effect in one of four cases (girls’ teacher-led).	Father smokes, mother smokes, sister smokes, best friend ever smokes, best friend currently smokes, believes most adults smoke, parental approval, peer pressure, perceived effects of smoking, country of birth, smoking intentions, smoking education classes.
Audrain-McGovern *et al.* [126], northern Virginia US, 2000–2003 (five waves), 14 years, 41%.	Four trajectories for smoking (9–12th grades). Latent class growth model. *Attrition analysis.*	Binary index for high- & low-receptivity (2 items: favorite brand, CBI). Receptivity is significant in 2 of 6 comparisons at 9th grade & 3 of 6 comparisons at 12th grade.	Gender, race, academic performance, alcohol use, marijuana use, depressive symptoms, novelty-seeking, peer smoking, physical activity, team sports participation.
Biener & Siegel [104], Mass. US, 1993 & 1997/98, 12–15 years, 58%. *Attrition analysis.*	Progression to smoking (100+ smokes in past 4 years) by baseline non-smokers. Logistic regression, but controls for only selected covariates. Unclear how these are selected.	Baseline receptivity to tobacco marketing (2 items: ownership of CBI, can name favorite ad’s brand). High receptivity is a predictor of progression to smoking, but moderate receptivity is not. High susceptibility is not significant if controlled for smoking susceptibility (p409). *Reports cross-tabulation for receptivity*.	Age, gender, race, parent education, household income, adult smoker in house, peer smoking, rebelliousness, depression, baseline initiation continuum, susceptibility to smoking.
Biener & Siegel [105], Mass. US, 1993 & 1997/1998, 12–15 years, 58%.	Eleven-point smoking initiation-susceptibility index (never smoked to 100 smokes & regular smoking past month). Multilevel regression.	Knowledge of tobacco slogans (12-pt scale) at follow-up (p207). Knowledge of tobacco slogans is a predictor of position on the smoking continuum, but omits other advertising covariates, including receptivity.	Age, gender, race, parent education, household income, peer smoking, adult smoker in house, perceived social value of smoking at follow-up, baseline initiation continuum. Mediation considered for perceived value but could be moderator relationship.
Charlton & Blair [106], 3 towns in northern UK, 1/1986 and 5/1986, 12–13 years, 100%.	Onset of smoking by gender for baseline nonsmokers. Stepwise logistic regression, but log-odds ratios not reported.	Cigarette-brand awareness; favorite advertisement; imputed TV sports cigarette-brand advertising. None of the advertising covariates are predictors of boys’ smoking onset (p815). For girls, awareness of at least one cigarette brand is significant.	Gender, parental smoking, peer smoking, positive view on smoking, negative view on smoking, perceived health effects of smoking, smoking education classes.
Choi *et al.* [107], California US, CTS, 1993 & 1996, 12–17 years, 49%.	Progression to established smoking (100+ smokes in past 3 years) by experimenters at baseline. Stepwise logistic regression.	Receptivity to tobacco advertising (3 items: own or willing to use CBI; have a favorite ad; could name any cigarette brand). Receptivity is a predictor of smoking at the high level, but not at the moderate level.	Age, gender, race, family relationships, family smoking, peer smoking, perceived peer smoking, perceived ability to quit, religiosity, school performance. Significant interactions between receptivity & other risk factors.
Dalton *et al.* [108], NH & VT US, 1999 & 2000/2001, 10–14 years, 73%.	Onset of smoking by baseline nonsmokers. Generalized linear (log-link) regression for relative risk ratios.	Smoking exposure in movies (for random sample of 50 movies). Receptivity to tobacco promotions is unreported covariate in multivariate regression. Movie smoking exposure is a significant predictor of onset.	Grade, gender, parent education, parenting style, school performance, parental smoking, sibling smoking, peer smoking, sensation seeking, rebelliousness, self-esteem, parents’ disapproval. Interactions.
Distefan *et al.* [109], California US, CTS, 1996 & 1999, 12–15 years, 67%.	Any smoking by baseline never-smokers. Popular stars’ movies in 3 years before baseline are reviewed. Logistic regression.	At baseline, respondents named their 2 favorite male & female movie stars. Favorite stars’ smoking predicts smoking for girls (but not boys). High receptivity also predicts smoking. *Reports cross-tabulation.*	Age, gender, race, school performance, family smoking, peer smoking, parents’ disapproval, susceptibility to smoking. Interactions with age, gender, *etc.*
Gidwani *et al.* [110], nationwide NLSY US, 1990 & 1992, 10–15 years, na.	Onset of smoking by baseline nonsmokers. Logistic regression.	TV viewing hours per day (0 to 5+ hours) at baseline. Statistically significant effects for 4-5 hours and more than 5 hours per day. Confidence intervals are unclear and some variable are excluded (p507).	Age, gender, race, math score, reading score, vocabulary score. Additional factors are household income, maternal education, mother’s age, maternal IQ, number of children in household.
Gilpin *et al.* [111], California US, CTS, 1993–1999, 1996–2002, 12–17 years & 18–23 years, 47% & 48%	Established smoking at follow-up by baseline experimenters and nonsmokers. Logistic regression. *Attrition analysis*	Receptivity to tobacco advertising at baseline (3 items: own or willing to use CBI; named highly advertised brand; name of brand in favorite ad). Receptivity is significant at moderate and high levels for both cohorts	Age, gender, race, school performance, parental smoking, sibling smoking, peer smoking, baseline smoking status. Interactions between receptivity and smoking status, peer smoking
Hanewinkel *et al.* [81], Schleswig-Holstein DE, 2005 & 2006, 10–16 years, 80%.	Onset of smoking by baseline never-smokers. Generalized logistic (log-link) regression and path analysis model.	Frequency of exposure to movies or videos that are rated as appropriate for ages 16 and older (FSK-16 rating). Significant results for two higher levels of viewing FSK-16 movies.	Age, gender, school performance, school type, parental smoking, sibling smoking, peer smoking, parenting style, sensation seeking.
Hanewinke & Sargent [122], Schleswig-Holstein DE, 2005 & 2006, 10–16 years, 82%.	Any smoking at follow-up by nonsmokers at baseline. Generalized linear model (log link), with school type as cluster variable.	Frequency of exposure to smoking in 50 popular US movies (extrapolated from 398 films); favorite tobacco ad. Significant results for movie exposure quartiles & favorite tobacco ad. *Reports cross-tabulation.*	Age, gender, school performance, school type, parental smoking, sibling smoking, peer smoking, parenting style, sensation seeking. Interactions between exposure & age, gender, *etc.*
Jackson *et al.* [112], North Carolina US, 2002 & 2004, 12–14 years, 85%. *Attrition analysis.*	Onset of smoking by baseline nonsmokers. Stepwise logistic regression, with separate results for blacks and whites.	Exposure to movies by rating; TV set in bedroom; hours of TV use; frequency of TV use; parental program rule for TV. In final model, R-rated movies & private TV are significant for whites. No variables are significant for blacks.	Grade, gender, race, school grades, parents’ education, family smoking, peer smoking, parental engagement, parental relationship, college aspirations, sensation seeking.
Lopez *et al.* [113], Asturias ES, base & 3 follow-ups, 13–14 years, 64%. A*ttrition analysis.*	Progression to regular smoking (one per week) by baseline nonsmokers. Stepwise logistic regression.	Number of brands identified in 3 commonly displayed billboard ads at baseline. Significant effect of number of brands on regular smoking at 6, 12, & 18 month follow-up.	Age, gender, SES, family smoking, peer smoking, school. Other variables are missing full description (attitude, social influence, intentions to smoke). Interactions.
Pierce *et al.* [114], California US, CTS, 1993 & 1996, 12–17 years, 61%.	Susceptible to smoking (combines nonsmokers & experimenters). Logistic regression. *See [131] for attrition analysis.*	Receptive to tobacco advertising (3 items: own or willing to own CBI; have a favorite ad; named brand in favorite ad). Receptivity is significant at moderate and high levels.	Age, gender, race, school performance, family smoking, peer smoking. Interactions between exposure to smokers & susceptibility are not significant.
Pierce *et al.* [115], California US, CTS, 1996 & 1999, 12–14 years, 65%.	Onset of smoking by never-smokers at baseline. Logistic regression.	Receptive to tobacco advertising at baseline (3 items: own or willing to use CBI; have a favorite ad; named brand in favorite ad). Receptivity is positive if more-authoritative parents.	Age, gender, race, school performance, parental education, family smoking, peer smoking susceptibility to smoking, authoritative parenting style. Interactions with age & gender.
Pierce *et al.* [116], California US, CTS, 1996 & 1999, 12–15 years, 67%.	Experimented with smoking by never-smokers at baseline; susceptible to smoking Logistic regression.	Receptive to tobacco advertising (3 items: own or willing to use CBI; have a favorite cigarette ad; named brand in favorite ad). Neither moderate nor high receptivity predicts experimentation or susceptibility.	Age, gender, race, school performance, family smoking, peer smoking, susceptibility to smoking, curious about smoking at baseline. Interactions with age and gender (not significant).
Pucci & Siegel [117], Mass. US, 1993 & 1997/1998, 12–15 years, 59%. *Attrition analysis.*	Brand of initiation for experimenters; brand of regular smokers. Simple correlation analysis.	Individual exposure to brand-specific advertising in sample of 14 magazines (307 of 627 youth read one or more magazines in sample). Brand exposure is correlated with smoking.	Gender, race (only two covariates reported).
Sargent *et al.* [118], rural VT US, 1996 & 1997, 1998, 8–17 years, 66%. *Attrition analysis.*	Smoking status index on 0-5 scale (0 = never-smoker, 5 = 100+ cigarettes in lifetime). Logistic regression.	Own or willing to own CBI. Receptivity to CBI predicts progression on smoking index scale. Change in receptivity predicts progression in subsample. *Reports determinates of receptivity.*	Grade level, gender, school performance, parental education, family smoking, peer smoking, baseline smoking status, tobacco prevention program intervention.
Thrasher *et al.* [123], Cuernavaca & Zacatecas MX, 2006 & 2007, 11–14 years, 83%.	Smoking onset (past yr) & current smoker (past 30 days) by never-smokers at baseline. Logistic regression. *Attrition analysis.*	Exposure to movie smoking in standard list of 42 movies (minutes); own CBI. Mixed results for movie exposure & smoking onset. Movie exposure predicts current smoking. CBI insignificant for both outcomes.	Age, gender, school type, parental smoking, sibling smoking, peer smoking, parental approval, parenting style, sensation-seeking, self-esteem, TV in bedroom.
Titus-Ernstoff *et al.* [124], NH & VT US, 2002 & 2003 (3 waves), 9–12 years, 90%. *Attrition analysis.*	Onset of smoking by baseline nonsmokers. Poisson regression (relative risk ratios) for each wave of exposure.	Exposure to smoking in 50 movies (assessed at each wave). Baseline & later exposures predict smoking initiation.	Age, gender, race, school performance, parental smoking, peer smoking, sensation seeking, rebelliousness, self-regulation, self-esteem, parent education, maternal responsiveness, maternal monitoring.
Weiss *et al.* [119], California US, 2000, 2002 & 2003, 10–13 years, 80%.	Smoking susceptibility (combines nonsmokers & smokers). Multilevel model. *Attrition analysis.*	Exposure to pro-tobacco media (TV portrayals & displays at tobacco outlets). Pro-tobacco media predicts smoking susceptibility. *Reports cross-tabulation for exposure*s.	Gender, race, immigration status, acculturation status, anti-tobacco media exposure. Interactions with pro- & anti-tobacco exposure; interactions with race & acculturation.
Wilkinson *et al.* [125], Houston, TX US, 2001 & 2003 (4 waves), 11–13 years, 90%.	Experimentation with smoking (ever, new). Stepwise logistic regression.	Movie-smoking exposure in a sample of 50 movies. For experimentation (new), movie-exposure is significant for Mexican-born, but not US born. *Reports exposure means.*	Age, gender, country of birth, family smoking, peer smoking, acculturation, parental education, risk taking, anxiety, detention. Interactions with country of birth & acculturation.
Wills *et al.* [120], NH & VT US, 1999 & 2000/2001, 9–13 years, 69%. *Attrition analysis.*	Onset of smoking by baseline never-smokers. Structural model with movie exposure at baseline as exogenous variable.	Movie-smoking exposure (number of occurrences in sample of 50 movies). Movie exposure has an indirect effect on onset through increased affiliation with peer smoking as well as a direct effect. *Reports table of correlations.*	Age, gender, race, school performance, parental education, parental smoking, sibling smoking, peer smoking, maternal responsiveness, mother’s rules, rebelliousness, sensation seeking, self-esteem, baseline smoking status.
Wills *et al.* [121], nationwide US, 2003 & 2004, 10–14 years, 85%. *Attrition analysis.*	Onset of smoking (ever smoked) by baseline never-smokers. Structural model with movie exposure at baseline as exogenous variable.	Movie-smoking exposure (number of occurrences in sample of 50 movies). Movie exposure has indirect effects on onset through smoking expectancies and peer smoking as well as a direct effect. *Reports table of correlations.*	Age, gender, race, school performance, family structure, parental education, parenting style, household income, parental smoking, sibling smoking, peer smoking, rebelliousness, sensation seeking, self esteem, self control, baseline smoking status.
